# Deciphering of the Genetic Control of Phenology, Yield, and Pellicle Color in Persian Walnut (*Juglans regia* L.)

**DOI:** 10.3389/fpls.2019.01140

**Published:** 2019-09-20

**Authors:** Annarita Marrano, Gina M. Sideli, Charles A. Leslie, Hao Cheng, David B. Neale

**Affiliations:** ^1^Department of Plant Sciences, University of California, Davis, CA, United States; ^2^Department of Animal Science, University of California, Davis, CA, United States

**Keywords:** leafing date, harvest, yield, pellicle, lateral bearing, quantitative trait loci mapping, genome-wide association study, breeding

## Abstract

Yield, nut quality, and ability to adapt to specific climate conditions, are all important factors to consider in the development and selection of new Persian walnut (*Juglans regia* L.) varieties. The genetic control of these traits is still unknown in walnut, limiting the accuracy and rapidity of releasing new cultivars for commercial use. We studied the genetic architecture of five traits crucial for either marketing (i.e., yield, lateral fruit-bearing, and pellicle color) or selection of individuals with specific phenology (i.e., leafing and harvest date). By combining over 30 years of phenotypic data with genetic profiles generated using the latest Axiom™ *J. regia* 700K SNP array, we were able to identify and confirm major loci for all these traits. In particular, we revealed that a genomic region at the beginning of Chr1 controls both leafing and harvest date in walnut, consistent with the observed strong phenotypical correlation between these traits, and including candidate genes involved in plant development, leaf formation, and cell division. In addition, a large genomic region on Chr11 that includes genes with a central role in flowering control and shoot meristem growth underlies both lateral fruit-bearing and yield in walnut. We observed a more complex genetic architecture for pellicle color, strongly influenced by the environment (*h* *^2^* = 0.43). We identified two marker-trait associations on Chr6 and 7 for pellicle color, where genes encoding a UDP-glycosyltransferase or involved in the response to oxidation were found. In conclusion, by combining classical quantitative trait loci (QTL) mapping and genome-wide association mapping, we deciphered, for the first time, the molecular pathways controlling walnut phenology, yield, lateral fruitfulness, and pellicle color. Our findings represent a further milestone in the transition from conventional to genome-assisted breeding in Persian walnut.

## Introduction

Persian walnut (*Juglans regia* L.), native to the mountains of Central Asia, is widely cultivated in temperate regions extending from the Americas to Europe to Asia (Zeven and Zhukovski, 1975). It is a monoecious and deciduous tree, with male and female bloom occurring at separate times (heterodichogamy). Although self-fertile, the desynchronized bloom time between male and female inflorescences of this wind-pollinated species strongly promotes outcrossing (Germain, 1997). Some walnut trees produce pistillate flowers only from terminal buds of shoots. Others are capable of flowering also at lateral buds, a trait called “lateral bud fruitfulness” that is generally associated with higher yields in young trees (Germain, 1990). The walnut fruit consists of a kernel, pellicle, shell and husk. The embryo, which forms the kernel, first becomes apparent approximately seven weeks after pollination and increases in size within the husk cavity until late July. During this period, the shell forms and hardens. The pellicle, the seed coat that covers the kernel, is a tissue of maternal origin and is responsible for perceived kernel color. Between August and October, the husk, the outer fleshy tissue covering the nut itself, starts to split, indicating the fruit has reached maturity and is ready for harvest (Ramos, 1997).

Before the advent of plant breeding, genetic improvement of walnut proceeded unconsciously through selection and propagation of the best trees. Today these discoveries form the core of germplasm collections serving as the foundation of breeding programs across the world (McGranahan and Leslie, 2012; Pollegioni et al., 2017). One of the most significant walnut breeding programs is the Walnut Improvement Program of the University of California-Davis (UCD-WIP), which began in the late 1940s with the purpose of developing improved scion and rootstock cultivars for the California walnut industry (Tulecke and McGranahan, 1994). As with all the walnut breeding programs worldwide, the UCD-WIP aims to enhance the yield of kernels with desirable visual and taste qualities, while reducing input requirements (e.g., water and chemicals) and production costs (Leslie and McGranahan, 2014). In walnut, yield depends on precocity, tree age, the percent of pistillate flowers developing into nuts (percent set), nut size, and especially the number of pistillate flowers produced (Solar and Štampar, 2003; McGranahan and Leslie, 2012). Consequently, incorporation of lateral fruit-bearing, most commonly found in walnut trees from Western China and Central Asia, has been the focus of many walnut breeding programs (Bernard et al., 2018a). In California, the first cultivar identified with this trait was “Payne,” intensively used afterward as a source of lateral fruitfulness in the UCD-WIP (Forde, 1975).

Phenological traits, and how these fit with local climate conditions, are also an important consideration in cultivar selection, especially in the current scenario of climate change. Recent studies regarding the physiological responses of walnut to global warming indicate that average bud-break date has advanced in some regions [e.g., Slovenia (Črepinšek et al., 2009)], but has been delayed in others due to lack of chilling during winter [e.g., California (Pope et al., 2013)]. Therefore, some current walnut growing regions could become inappropriate for walnut cultivation in the near future, whereas areas currently considered too cold might develop a suitable climate for walnut growth (Gauthier and Jacobs, 2011). The UCD-WIP aims to release late-leafing cultivars, which are less susceptible to late spring frost and rain-related disease, particularly walnut blight (*Xanthomonas campestris* pv. *juglandis*). In California, the first widely-planted cultivar to leaf out is “Payne,” with a bud-break in mid-March, while the latest is “Franquette,” which leafs out in the second half of April (Forde, 1975). Unfortunately, a late-leafing profile is also typically associated with late harvesting. In California, harvest can begin in late August and continues until November. In recent years, the strong popularity of Chandler, a mid-late season cultivar, has squeezed a large proportion of walnut production into the later part of the harvest season in California, resulting in underutilization of equipment and drying capacity in the early fall (Leslie and McGranahan, 2014). Later harvesting walnuts are also more likely to encounter harvest delays and quality loss due to autumn rains (Ramos, 1997). For these reasons, the UCD-WIP also aims to breed cultivars with earlier harvest dates than cv “Chandler.”

Nut characteristics are clearly important when developing and selecting new varieties. Kernel color, nut size, kernel percentage, and shell thickness, are all essential criteria for walnut marketing (Ramos, 1997). Walnut kernels are graded mainly on color, with the lightest color being the most valuable. There is considerable variation among cultivars in the ability to produce extra-light kernels. Usually, late-maturing varieties tend to produce lighter kernels, probably because of the ripening occurring during cooler weather (Sibbett et al., 1974). The UCD-WIP has invested considerable effort into developing cultivars with valuable kernel color. Among these, “Chandler,” and more recently “Ivanhoe,” were both selected for their pearly, pale yellow kernels, and “Robert Livermore,” a cultivar exhibiting a red pellicle, was released in 2004 for specialty markets (McGranahan and Leslie, 2004; McGranahan and Leslie, 2011).

As we attempt to improve the efficiency of releasing improved cultivars, understanding the genetic control of yield, phenology, and nut quality traits is crucial to selecting superior genotypes accurately on a molecular basis. While these traits have been extensively studied in other crops (Castède et al., 2015; Marinoni et al., 2018; Larsen et al., 2019), few gene tagging studies have been published to date in walnut (Bernard et al., 2018b). Using F_2_ progeny of Chandler, Dvorak et al. (2016) identified two markers flanking the major genomic region on linkage group (LG) 11 which controls lateral fruit-bearing in walnut. Also, Kefayati et al. (2018) detected a major quantitative trait locus (QTL) for leafing time, accounting for 52.0–68.8% of the phenotypic variance in a “Chandler” × “Kaplan-86” F_1_ population. However, both these studies explored a limited genetic diversity and, therefore, lacked mapping resolution. Also, they did not provide candidate genes putatively underlying the investigated traits. Recently, the new Axiom^TM^ *J. regia* 700K SNP array, which comprises over 600K single nucleotide polymorphisms (SNPs), was released and used to genotype a collection of 1,284 walnut accessions from the UCD-WIP (Marrano et al., 2018). Availability of these hundreds of thousands of genetic profiles has allowed the reconstruction the UCD-WIP pedigree as well as association mapping studies for water use efficiency (Famula et al., 2019) and nut-related traits (Arab et al., 2019).

Here, we describe the utilization of these valuable genomic resources to dissect the genetic control of five important traits in walnut; leafing date, harvest date, yield, lateral bud fruitfulness, and pellicle color. In particular, we applied classical QTL mapping and association mapping to correlate the genotypic data generated for 896 walnut trees with the phenotypic records collected in the UCD-WIP across 30 years. By combining two gene-tagging approaches, we identified and confirmed major loci for all traits studied, revealing, for the first time, the molecular pathways controlling these phenotypes and providing a basis for genomics-assisted breeding in walnut.

## Materials and Methods

### Plant Material and Genotypic Data

The present research was based on 896 individuals of the UCD-WIP, which included several well-known walnut cultivars (e.g., Chandler, Howard, Tulare, Vina, Franquette), advanced selections, and 34 full-sib families generated from controlled crosses in years 2004 to 2010. The largest family was “Chandler” x “Idaho” (CRxID) with 312 seedlings. Remaining families averaged 23 individuals each (**Table S1**). All individuals were genotyped using the latest Axiom^TM^ *J. regia* 700K SNP array as described in Marrano et al. (2018). *Poly High Resolution* (PHR) SNPs were used to test genotype-phenotype associations. SNP probes (71-mer) were mapped onto the new chromosome-level walnut reference genome (v2.0) (https://www.hardwoodgenomics.org/Genome-assembly/2539069) to obtain their chromosome locations. Specifically, a chromosome position was assigned to an SNP when its probe aligned uniquely on the genome with 98% identity and an E-value < 1e^-20^ for more than 95% of its length. The SNP dataset was then filtered for minor allele frequency (MAF > 5%) and missing rate (<20%). The genotypic dataset for this study can be found at https://hardwoodgenomics.org/Genome-assembly/2539069.

### Phenotypic Data and Statistical Analysis

Historical phenotype data for leafing date (LefD), harvest date (HarD), lateral bearing (LTB), yield, and pellicle color were obtained from the UCD-WIP database. LefD is the time at which more than 50% of the terminal buds have begun to open. HarD is the time when 95% of the hulls have loosened from the shells. Both LefD and HarD were scored in Julian days. LTB is defined as the percentage of lateral buds flowering on elongated, 1-year-old shoots. Yield is based on visual evaluation with a score of 0 (no nut production) to 9 (highest yield). Pellicle color was scored using the official color chart of the Dried Fruit Association (DFA), where a score of one indicated extra-light, two—light, three—amber, and four—dark amber kernels. The averaged DFA score of 10 nuts per tree was then used as an individual record. Kinship relationships derived from the UCD-WIP pedigree (Marrano et al., 2018) was used to estimate individual breeding values (EBVs) and heritability per trait. The overall linear mixed model can be represented as

$$Y_{\mathrm{ijklm}}=\mu +{\text{a}}_{\text{i}}+{\mathrm{pe}}_{\text{i}}+{\mathrm{age}}_{\text{j}}+{\mathrm{year}}_{\text{k}}+{\mathrm{block}}_{\text{l}}+{\text{e}}_{\mathrm{ijklm}}$$

where *Y* *_ijklm_* is the m-th observation taken in the k-th year on the i-th tree of the j-th age class and located in the l-th block. In particular, *a* *_i_* is the additive genetic effect of the i-th tree on the trait, *pe* *_i_* is the permanent environment contribution to the phenotype of the i-th tree, and *e* *_ijklm_* is the residual term. The numerator relationship matrix was used to account for the covariance between additive genetic effects. A permanent environment effect was included in the model to account for the covariance between multiple observations from the same tree. For each trait, different models were fit, including both *a* *_i_* and *pe* *_i_*, always as random effects, while age, year and block as random or fixed in a variety of combinations. The best model was then selected based on the Bayesian Information Criterion (BIC). Due to the categorical nature of yield, LTB, and DFA score, generalized linear mixed models were fit, relating the observed ordered categories to an underlying binomial density through a probit (yield) or logit (LTB and DFA score) link function. All models were fit and resolved using the REML estimation method implemented in ASReml v4 (Gilmour et al., 2009). The narrow-sense heritability (*h* *^2^*) of each trait was then calculated as the ratio between the additive variance (σ_A_) and the phenotypic variance (σ_P_ = σ_A_ + σ_PE_ + σ_e_). Afterward, Pearson correlation value (R) between each pair of traits was investigated with R/GGally v1.4 (https://cran.r-project.org/web/packages/GGally/index.html). Also, Principal Component Analysis (PCA) based upon EBV values was performed using the built-in R function “prcomp.”

### Linkage Map Construction and QTL Detection

The double pseudo-testcross mapping strategy was applied to construct individual maps for each parent of the F_1_ family “CRxID” (312 individuals) (Grattapaglia and Sederoff, 1994). Markers with a distorted segregation ratio (*p-value* < 0.01) were removed. The linkage analysis was carried out using the R/ASMap package (Taylor, 2015), which utilizes the MSTmap algorithm (Wu et al., 2008) to cluster markers into linkage groups and find the optimal marker order within each linkage group in a very computationally eﬃcient manner. Markers with a p-value < 1e^-12^ were clustered together, and the recombination frequencies were converted into genetic map distances (centimorgans; cM) using the Kosambi mapping function (Kosambi, 1943). MapChart v2.32 software (Voorrips, 2002) was used for the graphical visualization of the linkage groups.

Both parental maps were used to identify QTLs controlling the five traits of interest, implementing individual EBVs as phenotypic value. The QTL mapping analyses were carried out in R/QTL v1.44.9 (Broman et al., 2003). Single-marker test was first performed, where the marker effect on a specific trait is estimated by a simple linear regression between the marker and the trait (Doerge, 2002). Then, simple interval mapping (SIM) was applied. This estimates the likelihood score of a putative QTL within intervals of adjacent pairs of linked markers (Lander and Botstein, 1989; Haley and Knott, 1992). Multiple QTL Mapping (MQM) was also performed, using as cofactors markers pre-selected by multiple regression and backward elimination (Jansen, 1993; Arends et al., 2014). The significant logarithm of odds (LOD) threshold (*p-value* = 0.05) for each trait was determined with genome-wide permutation tests (1,000 permutations; Churchill and Doerge, 1994).

### Genome-Wide Association Mapping

Five hundred eighty-four individuals, including all UCD-WIP families (except “CRxID”) and cultivars, were used to perform genome-wide association study (GWAS) for the five traits studied. GWAS was carried out by applying three different models implemented in R/GAPIT v3.0 (Tang et al., 2016): the Multiple Loci Linear Mixed Model (MLMM), the Settlement of Mixed Linear Model Under Exclusive Relationship (SUPER), and the Fixed and Random Model Circulating Probability Unification (FarmCPU). These three models incorporate different algorithms to increase the power of detecting significant marker-trait association while avoiding false-positives due to confounding factors (Segura et al., 2013; Wang et al., 2014; Kusmec and Schnable, 2018). Both the kinship matrix and principal components (PCs) were included to correct for familial relatedness and population structure. In particular, the number of PCs to include was defined using the “model.selection” function implemented in GAPIT. A quantile-quantile (Q–Q) plot was used to check if the model was correctly accounting for both confounding variables. P-value adjustment for multiple testing was performed, and the Bonferroni corrected critical p-value (= 3.67e^-8^), and False Discovery Rate (FDR ≤ 0.05) were used to identify significant marker-trait associations.

### Linkage Disequilibrium and Candidate Genes

We used the RefSeq walnut gene annotation (https://www.ncbi.nlm.nih.gov/genome/annotation_euk/Juglans_regia/100/) mapped onto the reference genome v2.0 to identify candidate genes for the studied phenotypes. We first inspected the LD landscape around the marker-trait associations identified in the same genomic regions by both QTL mapping and GWAS. By using the default method of confidence intervals implemented in Haploview v4.2 (Gabriel et al., 2002; Barrett et al., 2005), we defined LD blocks. The entire genomic region located between the extreme SNPs of these LD blocks was then further investigated to identify candidate genes for the studied traits. We also explored whether any of the SNPs found to be significantly associated, in either of the gene mapping analyses, were falling within genic regions.

## Results

### Phenotypic Data and Statistical Analysis

Phenotypic records for all traits were collected by the UCD-WIP from 1988 to 2017 on trees ranging in age from one to 17 and distributed across 46 blocks. Both LefD and HarD were normally distributed (**Figures S1A, B**) and were slightly moved earlier across the years of the breeding program (**Figures S2A, B**). Yield, DFA score, and LTB were, on the contrary, were categorical traits, with the latter resembling a binomial distribution (**Figures S1C–E**). Both yield and LTB have strongly increased in the UCD-WIP since 1988, while the average DFA score decreased by nearly one score (**Figures S2C–E**). To account for the impact of year, age and block on each trait, and to estimate the genetic component of phenotypic variation, the animal model, which is a linear mixed model that includes individual breeding values as explanatory variables (Wilson et al., 2010), was applied. Based on the BIC, the best linear mixed models for LefD and HarD included year, age and block as fixed effects, while for yield and LTB all variables were defined as random effects. For the DFA score, the best fitting model did not include age as an explanatory variable and considered all others as random effects. This implies that pellicle color is not affected significantly by tree age. The narrow-sense *h* *^2^* was high for all traits, except the average DFA score (*h* *^2^* = 0.43). LTB showed the highest *h* *^2^* at 0.98, followed by LefD (0.88), yield (0.77) and HarD (0.68).

Based on individual breeding values estimated considering the entire walnut collection (896 individuals), the average HarD was 268.4 Julian days, with UC-06-005-8 the earliest tree (251 JD) and Scharsch Franquette the latest (290 JD; **Table 1**). Leafing date ranged from 73 Julian days (UC-91-031-8) to 117 (Ronde de Montignac), for an average of 89 Julian days. The percentage of lateral buds flowering per individual was 73% on average, with more than half of the whole collection being lateral (30–99% of lateral bud flowering). The yield score averaged 5.1, with UC-10-025-13 the most productive (score 7.8) and seedling UC-06-033-62 showing the lowest yield score (1.1; **Table 1**). Idaho had the highest average DFA score for pellicle color (3.3), with mostly amber kernels, while UC-03-001-977 had the lowest DFA score (1.1), indicating its great genetic merit in yielding high rates of extra-light kernels (**Table 1**).

**Table 1:** Summary statistics for harvest date (HarD), leafing date (LefD), lateral fruit-bearing (LTB), yield and pellicle color (average DFA) in the association panel and the F1 family ‘Chandler x Idaho’ (CRxID).

Trait	*h* *^2^*	*AM panel*				*CRxID progeny*			
		Min	Mean	Max	SD	Min	Mean	Max	SD
HarD	0.68	251.21	266.56	288.91	6.84	261.54	271.39	279.42	3.10
LefD	0.88	73.20	88.31	117.35	7.05	83.07	91.38	100.89	4.78
LTB	0.98	0.00	0.95	1.00	0.13	0.04	0.32	0.93	0.30
Yield	0.77	1.20	6.00	7.90	1.60	1.20	3.50	5.30	0.80
Average DFA	0.43	1.42	1.98	3.29	0.25	2.02	2.40	2.82	0.13

**Figure 1:**
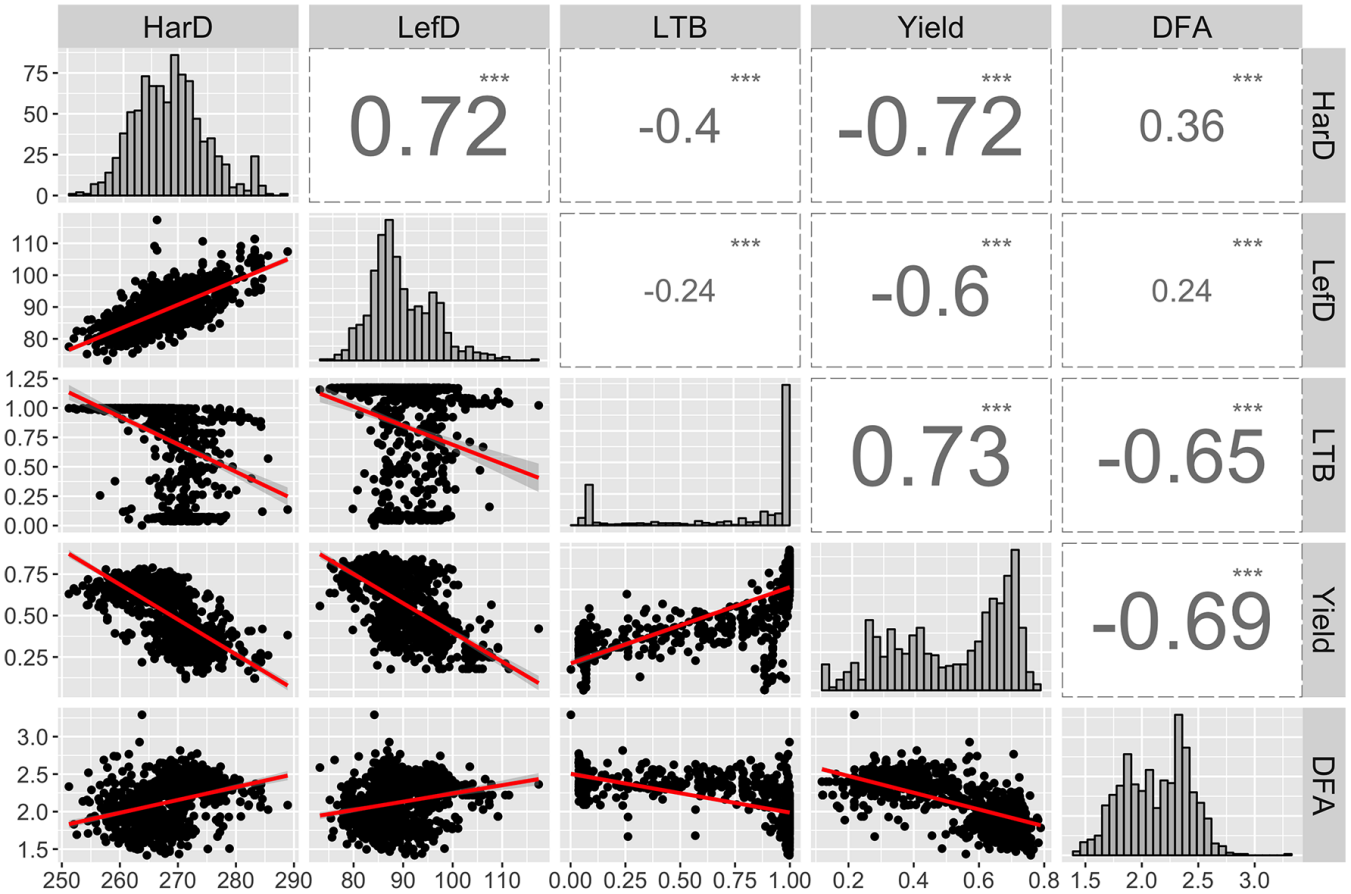
Distribution and correlation among the EBVs calculated for harvest date (HarD), leafing date (LefD), lateral bearing (LTB), yield, and pellicle color (DFA). ***p-value < 0.001.

We observed strong correlations among the main five target traits of the UCD-WIP. Specifically, LefD and HarD were positively correlated (*R* = 0.72; *p-value* < 0.001), suggesting that individuals with early leafing will also be early harvesting (**Figure 1**). Both phenological traits showed a high negative correlation (*R* = −0.6/−0.72; *p-value* < 0.001) with yield, suggesting higher yield would be expected in trees with early leafing and harvest dates. Also, the Pearson correlation analysis indicated that high-yielding individuals are generally lateral (*R* = 0.73; *p-value* < 0.001), and produce more extra-light kernels (*R* = −0.65; *p-value* < 0.001; **Figure 1**).

### Linkage Map Construction and QTL Detection

By combining the double pseudo-test cross strategy and the bin-based mapping approach, two parental linkage maps were constructed for the largest family CRxID. The female-backcross map (Chandler) spanned a total of 1,034.37 cM with 1,880 markers mapped into 16 LGs corresponding to the 16 walnut chromosomes (**Table 2**). The shortest linkage group (LG) was LG15 spanning 40.05 cM, which also contained the lowest number of SNPs at 21 and the largest region with no mapped markers (14.74 cM). Conversely, LG8 was the longest LG, spanning 112.58 cM, and had the highest number of SNPs mapped onto a single LG (227 SNPs). The average marker density in the female map was one SNP every 0.55 cM, with a total of five regions more than 10 cM without any molecular markers mapped (**Figure S3**). The male map (Idaho) contained 2,607 SNPs distributed into 16 LGs for a total length of 1,724.39 cM. The LG length ranged from 66.36 cM in LG9 to 148.87 cM in LG7, for an average LG length of 107.77 cM. The lowest number of SNPs mapped into a single LG was 101 in LG15, while the highest was 264 on LG9. The male map had five regions with no molecular markers mapped and exceeding the ten cM in length. The average marker density was slightly lower than that observed in the female map, with one SNP every 0.66 cM (**Figure S4**).

**Table 2:** Length and number of molecular markers per linkage group (LG) in the genetic maps of Chandler and Idaho.

*Chandler*			*Idaho*		
Linkage group (LG)	Length (cM)	SNP number	Linkage group (LG)	Length (cM)	SNP number
1	75.12	108	1	138.66	236
2	67.74	76	2	121.22	157
3	59.49	153	3	107.55	206
4	50.96	120	4	93.29	101
5	46.20	66	5	77.86	106
6	56.86	113	6	91.14	156
7	91.26	188	7	148.87	264
8	112.58	227	8	138.60	191
9	71.50	116	9	66.36	106
10	60.80	145	10	109.98	153
11	63.40	121	11	132.15	227
12	52.27	101	12	100.64	111
13	69.94	141	13	130.19	180
14	56.54	93	14	100.51	142
15	40.05	21	15	66.88	101
16	59.57	91	16	100.48	170
**Total**	**1034.27**	**1,880**	**Total**	**1724.39**	**2,607**

**Figure 2:**
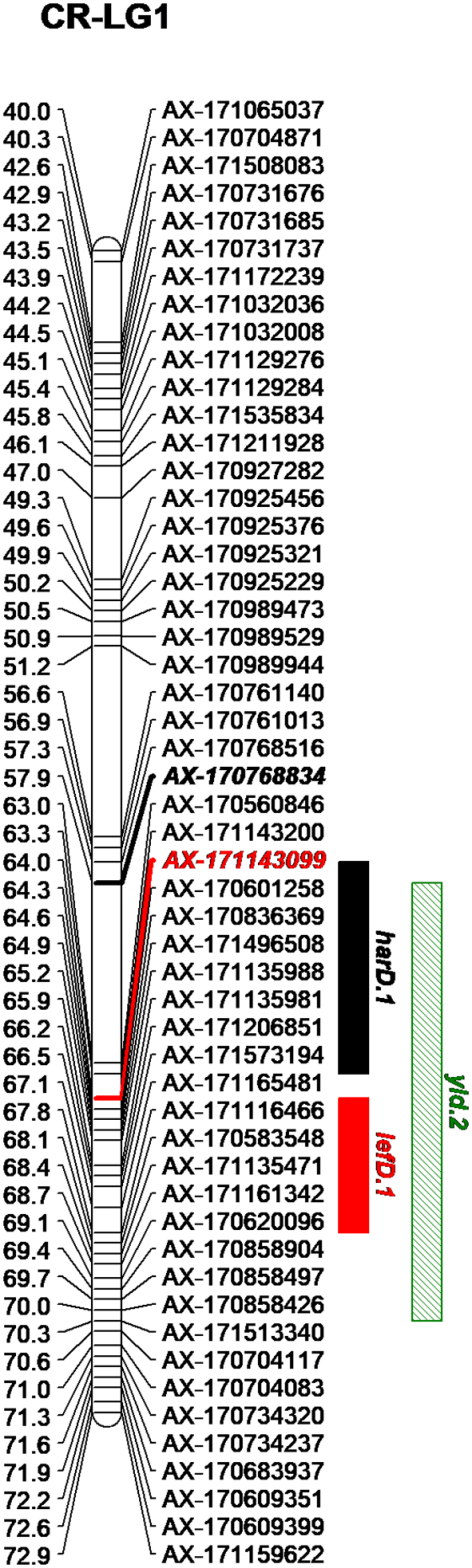
Genetic region on the LG1 of Chandler including the QTLs detected for harvest date (*harD.1*), leafing date (*lefD.1*) and yield (*yld.2*).

By integrating the phenotypic and genotypic data from the CRxID family, seven QTLs were detected for the phenological traits: five in Chandler and two in Idaho (**Table 3**). For LefD, one major QTL on LG1 in Chandler, explaining 81.97% of the total phenotypic variance, and one minor QTL on LG8 in Idaho, accounting for 7.53% of the phenotypic variation, were identified (**Figure S5**). Harvest date exhibited a more complex genetic architecture: four QTLs in Chandler on LG1, 5, 10 and 12 were detected, of which *harD.1* explained 16.78% of the phenotypic variation (**Figure S6**). In Idaho, an additional QTL on LG1 was detected for HarD, accounting for 7.85% of the trait variation (**Figure S6**). For yield and LTB, a major QTL in the same region on LG 11 of Chandler was identified, which explained 38.05% and 69.67% of the variation in nut productivity and lateral fruitfulness respectively (**Figures S7**, **8**). An additional minor QTL for yield was detected on LG1 in Chandler with only the multiple-QTL mapping analysis: this region of 12.43 cM also included *harD.1* and *lefD.1* (**Figure 2**; **Figure S8**), supporting the strong correlation among these three traits. Also, a minor QTL for LTB on LG6 of Idaho was detected, contributing to the phenotypic variation with the 7.51% (**Figure S8**). The QTL mapping analysis for the DFA score identified two QTLs only on the female map at LG6 and LG7, explaining totally 15.36% of the variability in pellicle color within the CRxID family (**Figure S9**).

**Table 3:** QTLs identified in Chandler and Idaho for pellicle color (dfa), harvest date (harD), lateral bearing (ltb), leafing date (lefD) and yield (yld). CI = one-LOD support interval. Underlined SNPs indicate multiple-trait associations.

QTL	LG	Peak (cM)	LOD (threshold)	CI (cM)	Var (%)	Marker	Position (BP)
***Chandler***							
*dfa.1*	6	21	2.97 (2.86)	10.83–45.07	4.29	AX-171029756	33,275,521
*dfa.2*	7	68	7.95 (2.86)	59.4–69.27	11.07	AX-171215261	44,165,465
*harD.1*	1	59	12.71 (2.98)	57.25–63.32	16.78	AX-170768834	7,756,021
*harD.2*	5	8.6	4.75 (2.98)	5.73–20.28	6.77	AX-170620990	19,761,618
*harD.3*	10	1.59	4.75 (2.98)	0.0–7.64	6.77	AX-170703758	1,010,797
*harD.4*	12	37.3	3.58 (2.98)	31.88–48.44	5.15	AX-170634283	26,038,393
*ltb.1*	11	45	80.3 (3.01)	44.61–45.88	69.67	AX-170876261	17,341,634
*lefD.1*	1	65	103 (3.08)	63.96–67.78	81.97	AX-171143099	4,650,186
*yld.1*	11	45.9	32.4 (3.05)	44.60–46.52	38.05	AX-170876261	17,341,634
*yld.2*	1*	65	5.59 (2.67)	57.89–70.32	1.48	AX-171143099	4,650,186
***Idaho***							
*harD.5*	1	19.9	5.52 (3.01)	12.18–21.51	7.85	AX-170916060	40,911,827
*ltb.2*	6	78	5.28 (3.22)	63.9–89.2	7.51	AX-170591244	3,978,361
*lefD.2*	8	136	5.42 (3.21)	126.1–138.6	7.53	AX-170703363	3,137,543

*detected only with multiple-QTL mapping.

### GWAS

**Figure 3:**
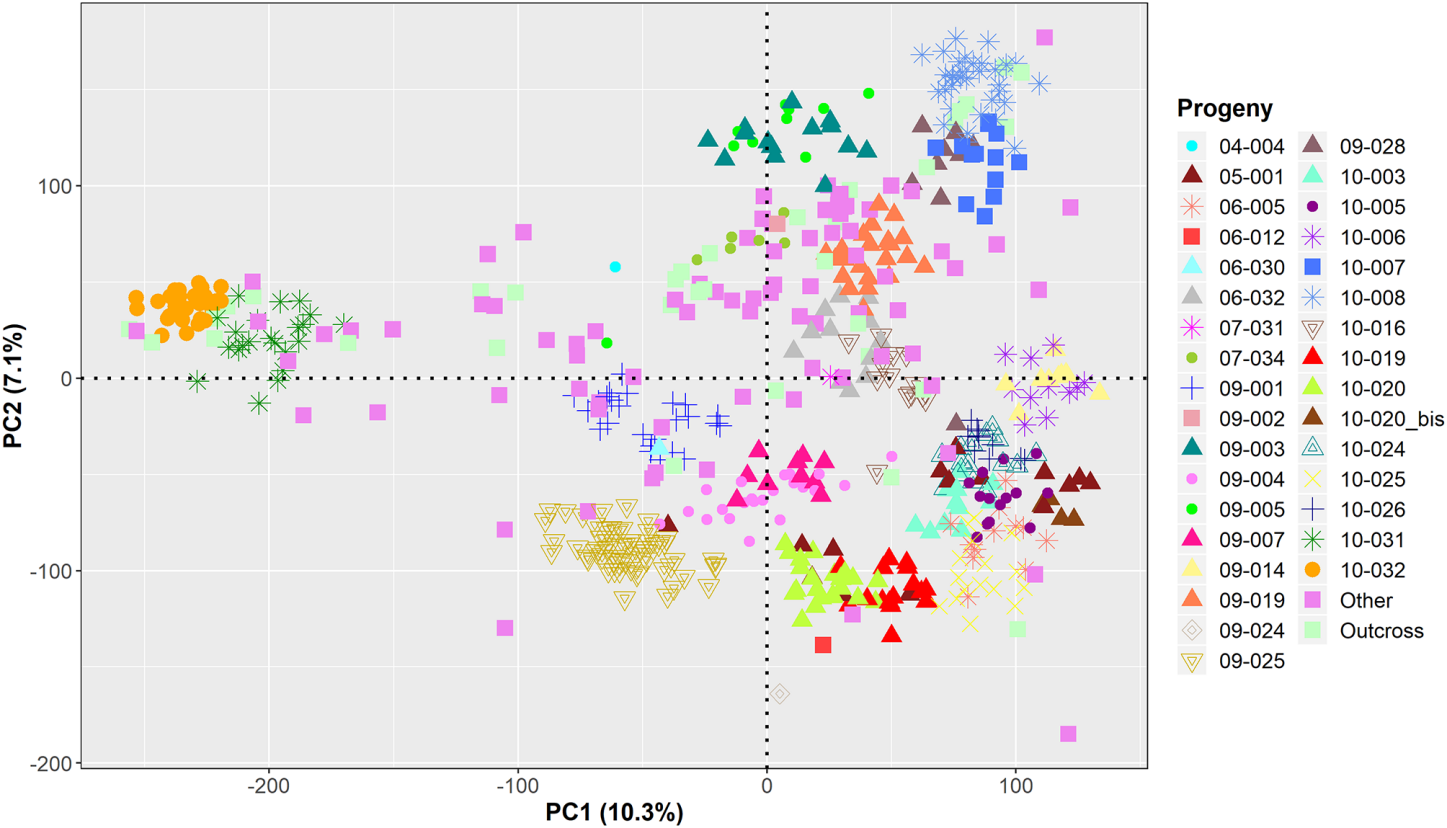
Projection of the genetic relationships among the 586 individuals used for GWAS onto the axes of the first two principal components (PCs).

GWAS was performed on the remaining 584 genotyped individuals, including well-known walnut cultivars and the smaller UCD-WIP families (**Table S1**). The filtered SNP panel used for GWAS included 266,224 SNPs with a physical chromosome position, and 6,436 variants mapping on unanchored scaffolds. For practical purposes, random positions on an extra LG17 were assigned to these unanchored SNPs. According to the model selection algorithm implemented in GAPIT v3.0, both the kinship matrix and PC1 were included for all traits, except LTB and average DFA score. The principal component analysis showed that major axes of variation reproduce the degree of relationship within the association panel (**Figure 3**).

**Figure 4:**
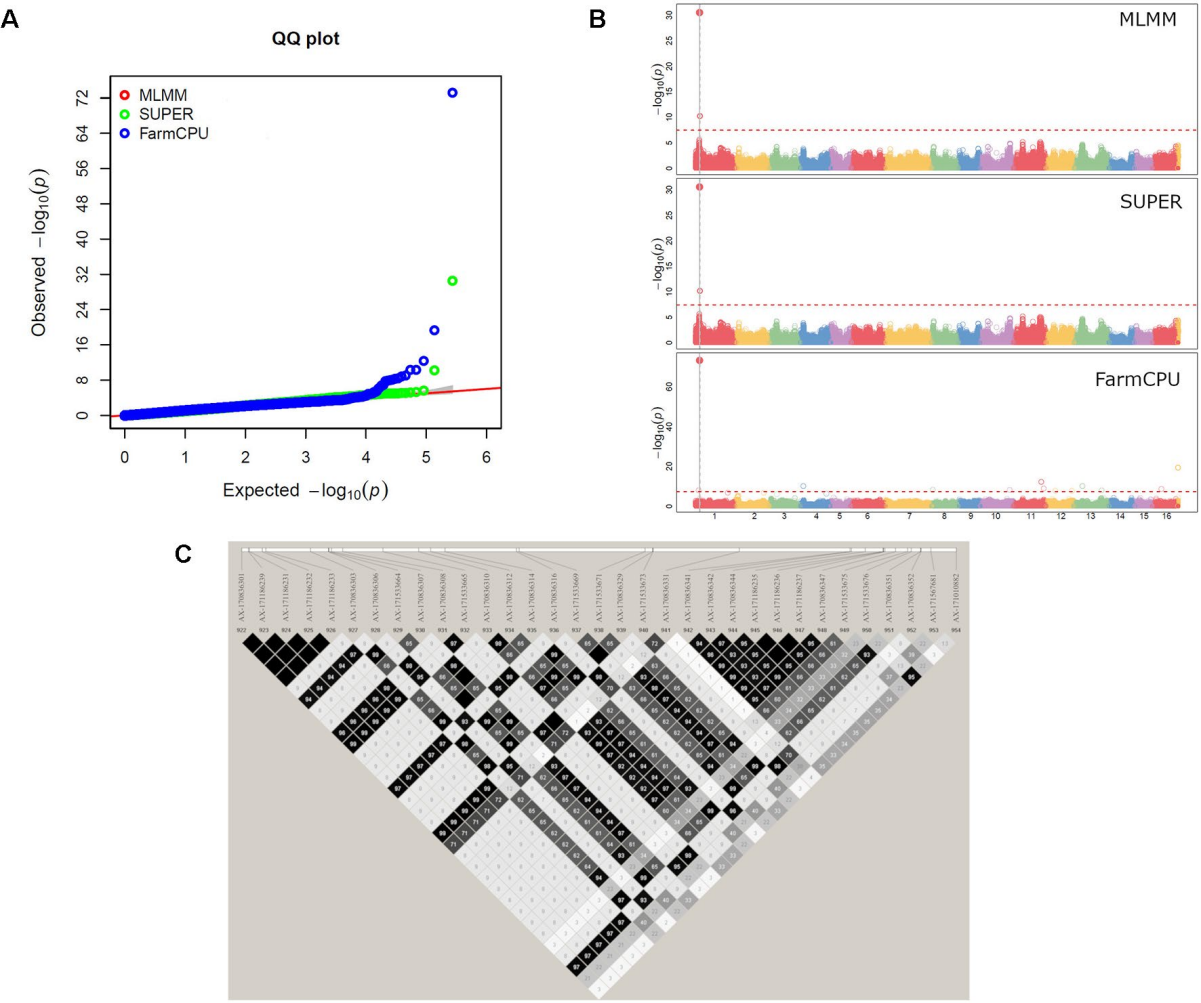
GWAS for leafing date in walnut: **(A)** Multiple QQ-plot and **(B)** Manhattan plot for the MLMM, SUPER and FarmCPU models; **(C)** LD block surrounding the marker AX-170836301 on Chr1.

The GWAS analysis identified 55 significant marker-trait associations. Lateral bearing yielded the highest number of significantly associated SNPs per trait, with three SNPs on Chr1, four SNPs on Chr4, one SNP on Chr8, one SNP on Chr9, seven SNPs on Chr11 and two SNPs on Chr13 (**Table S2**; **Figure S10**). The marker-trait associations on Chr11 covered a region of 25 Mb accounting for almost 90% of the trait phenotypic variance (R^2^ ranging from 0.01 to 0.31), and also included the major QTL *ltb.1* identified in the QTL mapping analysis. The trait with the second highest number of significant associations detected was LefD, with 14 SNPs found on Chr1, 4, 8, 10, 11, 12, 13, 16, and 17 (**Table S2**; **Figure 4**). Interestingly, the three SNPs identified on Chr1 spanned a genomic region of 1.6 Mb that also included the marker AX-171143099 flanking the mapped QTL *lefD.1* (**Figures 2**–**4**). The effect of an allelic substitution on LefD ranged from 1.09 Julian days at marker AX-170858858 to −7.32 at SNP AX-170836301. The association analysis for LefD also identified one SNP on Chr8 at 1.6 Mb from the marker AX-170703363 flanking the minor QTL *lefD.2*.

As observed in the QTL mapping analysis, two SNPs on Chr11 were significantly associated with yield and mapped closely to the genomic regions correlated with LTB (**Table S2**). These two SNPs were 1 Mb apart and explained more than 20% of the variability in yield in the association panel. Also, eight other significant marker-trait associations for yield were detected on Chr1, 2, 3, 6, 7, 13, 16, and 17 (**Table S2**; **Figure S11**). The GWAS analysis for HarD resulted in nine SNPs exceeding the Bonferroni *p-value* threshold: one on Chr1, one on Chr3, one on Chr10, one on Chr11, three on Chr13 and one on Chr16 (**Figure S12**). The marker AX-170770379 on Chr1 was 1.2 Mb apart from the QTL *harD.1*, confirming the central role of this genomic region in determining the harvesting season of walnut in California (**Table S2**). Also, for pellicle color, the GWAS analysis identified two SNPs in the proximity of the two QTLs *dfa.1* and *dfa.2*. The former was at 2.3 Mb from the locus AX-170725746 on Chr6, while the latter was 6.8 Mb from the most significant association for the DFA score on Chr7. Two more SNPs on Chr9 and 12 resulted significantly associated with the DFA score (**Table S2**; **Figure S13**). They all had a small effect on the phenotype, highlighting the complex genetic architecture of pellicle color in walnut.

### LD Blocks and Candidate Genes

Based on the QTL mapping and GWAS results, it can be inferred that there are major QTLs on Chr1 for HarD, on Chr1 and Chr8 for LefD, on Chr11 for LTB and yield, and on Chr6 and 7 for the DFA score. The active selection for these traits across the years in the UCD-WIP may have extended the LD surrounding the target loci. Thus, we investigated the LD extent around the most significant marker-trait associations and defined LD blocks to search for candidate genes involved in the five traits of interest (**Tables S3**, **4**).

The two SNPs on Chr1 associated with HarD were not in LD to each other (*r* *^2^* = 0.03) and fell in two different LD blocks: the marker AX-170770379 formed a 5-kb LD block together with other two SNPs and was in strong LD with the most adjacent LD block, including 10 markers and spanning 25 kb (**Figure S14**). In this genomic region (both 5–25 kb blocks), one candidate gene (LOC108987959; **Table S4**) was identified, coding for a TPX2-like protein and located 1 kb apart from SNP AX-170770379. The marker AX-170768834, flanking the QTL *harD.1*, belonged to a small LD block (<1 kb) where no genes could be identified (**Table S3**).

We also defined four LD blocks around the most significant SNPs on Chr1 associated with LefD. These were not in LD to each other (*r* *^2^* = 0.03–0.35), except for the SNPs AX-171143099 and AX-170560823, which were moderately correlated (*r* *^2^* = 0.53). The marker AX-170858858 formed a small LD block (< 1 kb) without any annotated candidate genes (**Table S3**). The SNP AX-170836301, which had a MAF of 0.1 in the association panel, fell within an LD block of 71 kb (**Figure 4**), where 11 candidate genes were identified (**Table S4**). Within this 71-kb LD block, it was observed that the marker AX-170836301 was in strong LD (*r* *^2^* = 1) with four other SNPs, organized into only two haplotypes, GTCGG (frequency = 0.9) and TGTAC (frequency = 0.1). The other two most significant SNPs AX-171143099 and AX-170560823 fell in blocks of 43 and 13 kb, respectively. In the 43-kb block, four genes were identified (**Table S4**). Marker AX-171143099 fell within the locus LOC108984557 coding for an ABC transporter. The 13-kb LD block included three genes, all encoding a chloroplast-like allene oxide cyclase. For LefD, a QTL on Chr8 was also detected with both gene-mapping approaches. While it was not possible to define any LD block for the SNP AX-170703363, due to its low MAF (<0.05) in the association panel, an LD block of 3 kb around the marker AX-170702771 was observed (**Figure S15**). Here it was found the locus LOC109001757 encoding the probable indole-3-pyruvate monooxygenase YUCCA4 (**Table S4**).

Defining LD blocks was more complicated for LTB since the six most significantly associated SNPs covered a large genomic region of 12.5 Mb. Also, three SNPs (AX-170908273, AX-170909006, AX-170960527) were in Hardy-Weinberg disequilibrium (*p-value* < 0.01). Six LD blocks of 75, 464, 49, 14, 300, and 151 kb were identified (**Table S3**). In these blocks, 91 candidate genes were found, and the SNPs AX-170722086 and AX-171205312 fell in two of them (**Table S4**). Also, Chr11 was searched for LD blocks around the three most significant marker-trait associations identified for yield; two LD blocks of 380 and 233 kb, respectively, were found, which included 45 candidate genes for yield (**Tables S3**–**5**), 35 of which were also identified for LTB.

For the DFA score, the LD extent was investigated in the two genomic regions on Chr6 and Chr7. On Chr6 one block of 51 kb around SNP AX-171029756 (**Figure S16**) and another block of 3 kb around the marker AX-170725746 were found. Both SNPs fell in genic regions: the former was located within the loci LOC108986141 and LOC109019067, encoding the DEAD-box ATP-dependent RNA helicase 27-like and the DNA-binding protein REB1 respectively, while the latter was found within the gene LOC108985377 (**Table S4**). In the genomic region on Chr7 associated with the DFA score, an LD block (17 kb) was found only for the SNP AX-170832900, which included four candidate genes coding for glycosyltransferases and heat-shock proteins (**Table S3**-**4**).

Aside from searching for candidate genes in the genomic regions surrounding the marker-trait associations detected with both gene-mapping methods, we also investigated whether any of the other SNPs identified as associated in either QTL mapping or GWAS fell in coding regions. It was found that, for HarD, the marker AX-170620990 on Chr5 and the SNP AX-171186285 on Chr11 were located within two genic loci (LOC109007348 and LOC108992128, respectively). Eight SNPs significantly associated with LTB fell in nine genes, encoding enzymes and transporters involved in carbohydrate metabolism processes, cellular signaling and ATP hydrolysis (**Table S4**). Also, for LefD, six SNPs fell in six candidate genes, codifying for proteases, oxidase, and acetylglucosaminyltransferase (**Table S4**). In addition, five of the trait-associated SNP loci identified in either one of the two gene-tagging methods caused a non-synonymous substitution in the protein coding sequences (**Table S4**).

## Discussion

For the first time in walnut, we dissected the genetic basis of five traits critical either for walnut processing and marketing, such as yield, lateral bearing, and pellicle color, or for the selection of individuals with specific timing of phenological events (i.e., leafing and harvest dates). Our study represents a further achievement in a long-term project of bringing molecular breeding in the UCD-WIP, which started with the release of the walnut reference genome (Martínez-García et al., 2016) and moved to the whole-genome resequencing of the UCD-WIP founders (Stevens et al., 2018) to the development of the high-density Axiom^TM^ *J. regia* 700K SNP array (Marrano et al., 2018). Here we present a new milestone in walnut genetics, as a source of inspiration for the introduction of genomics-assisted breeding in tree nut crops.

### Trait Heritability and Correlation

We estimated the genetic components for the five traits of interest by using the historical phenotypic data recorded within the UCD-WIP over 30 years. Thus, since these phenotypic data were initially not collected for any genetic mapping purpose, they were extremely heterogeneous in terms of years, age and localities. However, historical phenotypic data have been successfully used for gene mapping studies in other tree crop species, such as apple (*Malus domestica*; Larsen et al., 2019) and grape (*Vitis vinifera* L.; Migicovsky et al., 2017). Martínez-García et al. (2017) used a collection of over 15 thousand walnut accessions to predict the breeding values and genetic components of HarD, yield, LTB and the ratio of extra-light kernels produced, based on the historical UCD-WIP pedigree. Our 896 individuals were a subset of this large collection and were part of the new, more accurate UCD-WIP pedigree, recently reconstructed using high-density molecular markers (Marrano et al., 2018). The availability of a pedigree with newly discovered relationships and cleaned from any recording errors can ensure more accuracy in the estimation of the genetic components for the traits of interest. This can explain the discrepancies between our narrow-sense *h* *^2^* estimates and those observed by Martínez-García et al. (2017). In particular, we observed a higher value of *h* *^2^* for LTB and yield, along with a more substantial proportion of lateral bearing individuals (30-100% of lateral bud flowering) and a higher average yield score. The value of *h* *^2^* for HarD (0.68) was smaller than the one obtained by Martínez-García et al. (2017), but accession UC-06-005-8 was found to be the earliest in both studies. This consistency regarding the genetic merit of UC- 06-005-8 for HarD makes this genotype a good candidate for advancing the harvesting season in California by almost 1 month (September 8^th^) relative to the cv. Chandler (October 6^th^). We also estimate that 88% of the variability of LefD in our collection is heritable. A UCD-WIP goal is to release late leafing cultivars to avoid walnut blight, thus reducing the need for control measures (Ramos, 1997). The latest leafing genotypes was “Ronde de Montignac,” along with other French varieties such as “Scharsch Franquette” and “Fernor.” Overall, the high values of *h* *^2^* observed for all traits suggest that effective and accurate genetic improvement can be predicted for these phenotypes under specific selection schemes (Wilson et al., 2010). This does not apply to pellicle color; whose phenotypic variance was only for 43% heritable. The same was observed by Martínez-García et al. (2017) for the ratio of extra-light kernels produced, confirming the complex genetic structure of this trait and its strong dependence on the environment. Orchard structure, tree physiology and microenvironmental conditions occurring within a tree canopy are all factors likely contributing to the variation of pellicle color in walnut. Lampinen et al. (2007) observed that, within a tree canopy, yellow or black kernels can occur in the inner canopy at shaded positions, due to photosynthate restrictions related to lack of light or leaf loss. In our correlation analysis, average pellicle color was negatively correlated with yield and fruit-bearing habit (**Figure 1**). This result is encouraging for breeding since selection for genotypes with low average DFA scores (high percentage of extra-light or light kernels) will also target lateral fruiting, and thus, highly productive individuals. Multiple-trait selection will also be possible for HarD and yield: individuals with early harvest dates can also be candidates for high yield. On the contrary, the selection for both late leafing and early harvesting individuals will be more complicated due to their positive correlation. Selection of late-leafing seedlings will likely also shift harvest dates later (Forde, 1975).

### Gene Mapping Strategies

Due to this intricate pattern of relationships between the five studied traits and the strong influence of the environment on some of them, deciphering their genetic control is fundamental to assisting walnut breeders in the rapid development and introduction of improved cultivars. To do so, we combined the power of QTL mapping with the high resolution of GWAS. We first ran classical QTL mapping for “CRxID,” the largest family of our genotyping panel, derived from a cross of two parents with very different profiles in many traits, especially yield and nut quality. Chandler is one of the most popular walnut cultivars worldwide because it is highly lateral fruitful, relatively late leafing, and produces extra-light, pale yellow colored kernels (Tulecke and McGranahan, 1994). In contrast, “Idaho” is an early leafing and terminal-bearing individual, characterized by low yield and very large nuts of poor quality, as confirmed by its DFA score, the highest observed in our analysis (**Table 1**). The two parents also showed differences in the length and marker density of their genetic maps, with the Chandler linkage map being shorter and denser in molecular markers than that of Idaho. A short female linkage map was also developed in previous works (Luo et al., 2015; Kefayati et al., 2018), suggesting the possibility of a low recombination rate in the female in walnut (Trivers, 1988).

Even though the selection of parents at the extreme ends of a phenotypic trait increases the chance of detecting QTLs, QTL mapping has limited mapping resolution and relatively low accuracy in estimating the size of QTLs (Xu, 2003; Collard et al., 2005). In addition, the effect of the identified QTL can change in different genetic backgrounds due to interaction with other loci, recombination, or epistasis (Holland, 2001). For these reasons, we decided to perform GWAS in a broader genetic background to validate the QTLs identified with the classical QTL mapping analysis. The association panel included 33 families, derived by crossing 32 parents, including Chandler, which was the female parent of 22 individuals and the male parent of three samples. The 32 parents were related to each other by first and second-degree relationships and also displayed a genetic structure according to their geographical provenance (Marrano et al., 2018). The association panel showed considerably more phenotypic variability than the progeny “CRxID” for all traits except LTB. Almost all individuals were lateral bearing, highlighting the positive genetic gain for this trait in the UCD-WIP.

The application of GWAS allowed to confirm all major QTLs found with classical QTL mapping, and to identify additional marker-trait associations for all five traits, highlighting their polygenic nature. Twenty trait-associated SNPs accounted for low phenotypic variance (R^2^ ∼ 0) (**Table S2**). This may be related to the low MAFs observed for some of them (MAF ≤ 015), suggesting the effect of rare alleles not well represented within the breeding program (Ingvarsson and Street, 2011). Another explanation can be found in the relationships between the trait-associated SNPs and the confounding factors (i.e., familial relatedness and population structure), which could weaken the real associations (Liu et al., 2016). However, the identification of even loci accounting for small amounts of the total phenotypic variation in our association panel, supports the robustness and power of the GWAS models applied.

### A Genomic Region at the Beginning of Chr1 Controls Phenology in Walnut

Both gene tagging strategies detected major QTLs for leafing and harvest date at the beginning of Chr1 (**Figures 2**–**4**, **S12**), suggesting a possible common genetic pathway may control these two traits which strongly correlated with each other (**Figure 1**).

During the spring, a complex signaling network mediated by plant hormones and transcription factors activates dormancy release, ending with bud break in response to warm temperatures (Falavigna et al., 2019). At this stage of plant awakening, plant pathogens become active as well, exposing the plant to the risk of serious diseases, such as walnut blight. In this regard, we identified candidate genes for LefD with functions related to pathogen defense (protein LYK2-like gene (Gu et al., 2017), and biosynthesis of plant hormones, as jasmonic acid (allene-oxide cyclase genes) and auxin (YUCCA4 gene), reported to have a role in plant development, response to stress and leaf formation (Cheng et al., 2007; Stenzel et al., 2012). Other candidate genes identified for LefD are involved in leaf cuticle formation (ABC transporter I family member 11 gene (Leng et al., 2014), as well as in the regulation of leaf senescence and the response to phosphate starvation (the WRKY transcription factor 75 gene (Bakshi and Oelmüller, 2014).

About 1.7 Mb downstream from the candidate loci on Chr1 for LefD, we identified the protein TPX2-like gene as likely involved in HarD in walnut. The TPX2 protein is required for spindle assembly during mitosis (Wadsworth, 2015). Walnut fruit reaches maturity by a two-stage development process. During the first phase, cell divisions cause an increase in size and weight of the entire fruit, but the embryo accelerates its growth mostly during the second phase of fruit development (Ramos, 1997). Auxin regulates many aspects of plant growth and development, including embryogenesis, through a family of functionally distinct DNA-binding auxin response factors (ARFs; Li et al., 2016). We identified an auxin response factor 4-like gene on Chr11 as a candidate locus for HarD. This gene specifically codifies for ARF4, whose involvement in fruit development has been demonstrated in tomato (Sagar et al., 2013). Therefore, the central role of both the TPX2 and ARF4 proteins during cell division and fruit development makes them strong candidates for HarD regulation in walnut.

### Genes Controlling Flowering and Shoot Meristem Proliferation are at the Basis of Lateral Bearing and Yield in Walnut

The strong selection exerted in favor of LTB and yield within the UCD-WIP during many years has created a large LD block on Chr11, where lack of recombination makes it difficult to select the best candidate gene for these two traits with precision.

Within this large genomic region, we identified genes with a central role in flowering control and shoot meristem proliferation (**Table S4**; Castède et al., 2015). For instance, we identified an auxin efflux carrier component 5 gene, which regulates intracellular auxin homeostasis and metabolism. In addition to controlling fruit development, auxin is a crucial regulator of flowering in plants. It determines the site of flower initiation, controls floral organ growth and the pattern of formation within a floral organ, and regulates floral meristem cell proliferation (Li et al., 2016). We also found three genes encoding the *FLOWER LOCUS T*-INTERACTING PROTEIN 1 (FTIP1), a key regulator of the transport of the FT protein from leaves to shoot apical meristems (SAM) for the initiation of flower development in response to day length and photoperiod (Liu et al., 2012). Another key regulator of the circadian clock and flowering time is the transposase-derived transcription factor FAR-RED IMPAIRED RESPONSE1 (FAR1; **Table S4**), which has been shown to have a central role also in shoot meristem determination and flower development (Ma and Li, 2018).

The maintenance and size of shoot meristems are regulated by an elaborate genetic control that involves a variety of transcription factors and receptors. Receptor-like protein CLAVATA2 is a part of this feedback loop. Its mutant in maize exhibited fasciation and high yield, with increased proliferation of ear inflorescence meristem and modest alteration in floral meristem size and organ number (Taguchi-Shiobara et al., 2001). For LTB and yield, we identified two genes encoding the CLAVATA2 protein, together with a genic locus coding for the protein FANTASTIC FOUR 4 (FAF4), which modulates shoot growth in *A. thaliana* (Wahl et al., 2010).

Future fine-mapping strategies will be fundamental to identify which among the 91 genes detected here are the true causative candidate genes for LTB and yield in walnut (Dvorak et al., 2016). Also, the high correlation between yield and LTB might mask the detection of additional QTLs for yield, leaving genetically unexplained more than 50% of yield variability within the UCD-WIP. New quantitative phenotyping protocols for yield as well as gene tagging studies for additional traits on which yield relies (e.g., percent set, nut weight), will help to dissect the genetic control of yield in walnut further.

### Candidate Genes for Pellicle Color in Walnut

We also undertook the first study of the genetic architecture of pellicle color in walnut. This quality trait has high marketing importance since light-colored kernels command a higher price in the market. Colaric et al. (2005) studied the phenolic composition of ripe fruits in ten walnut cultivars and observed that most of the pellicles had much higher contents of phenolics than the kernel. This natural richness in phenolic compounds of the pellicle, which represents only 5% of the fruit weight, confers to this thin cover a protective role against fatty acid oxidation. Therefore, underpinning the genetic basis of pellicle color in walnut is important not only for marketing purposes but also to understand the mechanisms behind the antioxidant activity and nutritional value of walnut.

We found the LOC108991777, encoding a UDP-glycosyltransferase (**Table S4**), as a candidate gene involved in walnut pellicle color. The superfamily of UDP-glycosyltransferases includes enzymes that catalyze the addition of glycosyl groups from a UTP-sugar to a small hydrophobic molecule, and are involved in the biosynthesis of many plant metabolites, including anthocyanin pigments (Li et al., 2001). Genes for UDP-glycosyltransferases have also been identified as involved in red pear skin color (Kumar et al., 2019). We also observed that the most significant SNP associated with the DFA score on Chr6 fell within the LOC108986141, encoding a DEAD-box ATP-dependent RNA helicase 27-like. The DEAD-box RNA helicases participate in every aspect of RNA metabolism. In rice (*Oryza sativa*), a DEAD-box helicase ATP-binding protein (OsABP) was upregulated in response to multiple abiotic stress treatments, including blue and red-light exposition (Macovei et al., 2012). Thus, it is possible that the DEAD-box ATP-dependent RNA helicase identified in our analysis is involved in the protection against oxidation of walnut pellicle.

## Conclusions

By combining classical QTL mapping and GWAS, we were able to identify and confirm major genomic regions for all traits in our study. Using these two complementary approaches of gene tagging, each with different levels of resolution and power, we contained type I error (false-positive) related to the use of EBVs as phenotypes for genetic mapping (Ekine et al., 2014). The identification of genotype-phenotype correlations in the same regions by both methods implies the true involvement of these genomic regions in the phenotypes of interest. At the same time, we account for familial relatedness and population structure by applying new, more efficient and more powerful AM models, noted to reduce type II error (false-negative; (Tang et al., 2016). Our comprehensive analysis provides, for the first time, candidate genes for leafing and harvest dates, fruit bearing, yield, and pellicle color in walnut. The validation of our marker-trait associations in newly available materials within the UCD-WIP, as well as in foreign walnut collections, will help to set-up marker-assisted breeding for these five important traits in Persian walnut. Also, new efforts need to be invested in improving phenotyping strategies, in order to quantify accurately and objectively the phenotypic variation within breeding programs. New automated phenotyping methods are being explored and initiated within the UCD-WIP, especially regarding pellicle color (Sideli et al., 2019). Integration of these new phenotyping strategies will be fundamental to further expand our knowledge regarding the genetic control of phenology, yield and kernel quality in walnut.

## Data availability

The genotypic dataset for this study can be found at https://hardwoodgenomics.org/Genome-assembly/2539069

## Author Contributions

AM coordinated the research, performed all the genetic analysis, and wrote the manuscript. GS assisted AM in analyzing and interpreting the data of pellicle color. CL provided the phenotypic data from the UCD-WIP database and his experience in walnut breeding to interpret the genetic results. HC contributed to fit the animal models for estimating breeding values and heritability. DN conceived and coordinated the research. All coauthors revised the manuscript.

## Funding

HC’s work is support by US Department of Agriculture, Agriculture and Food Research Initiative National Institute of Food and Agriculture Competitive Grant No. 2018-67015-27957.

## Conflict of Interest Statement

The authors declare that the research was conducted in the absence of any commercial or financial relationships that could be construed as a potential conflict of interest.
