# Supplementary material for: Deciphering of the Genetic Control of Phenology, Yield, and Pellicle Color in Persian Walnut (Juglans regia L.)
Source: Front Plant Sci. 2019 Sep 20;10:1140. doi: 10.3389/fpls.2019.01140 (PMC6764078; doi:10.3389/fpls.2019.01140)
Supplement: Supplementary file 1 [file DataSheet_1.pdf]

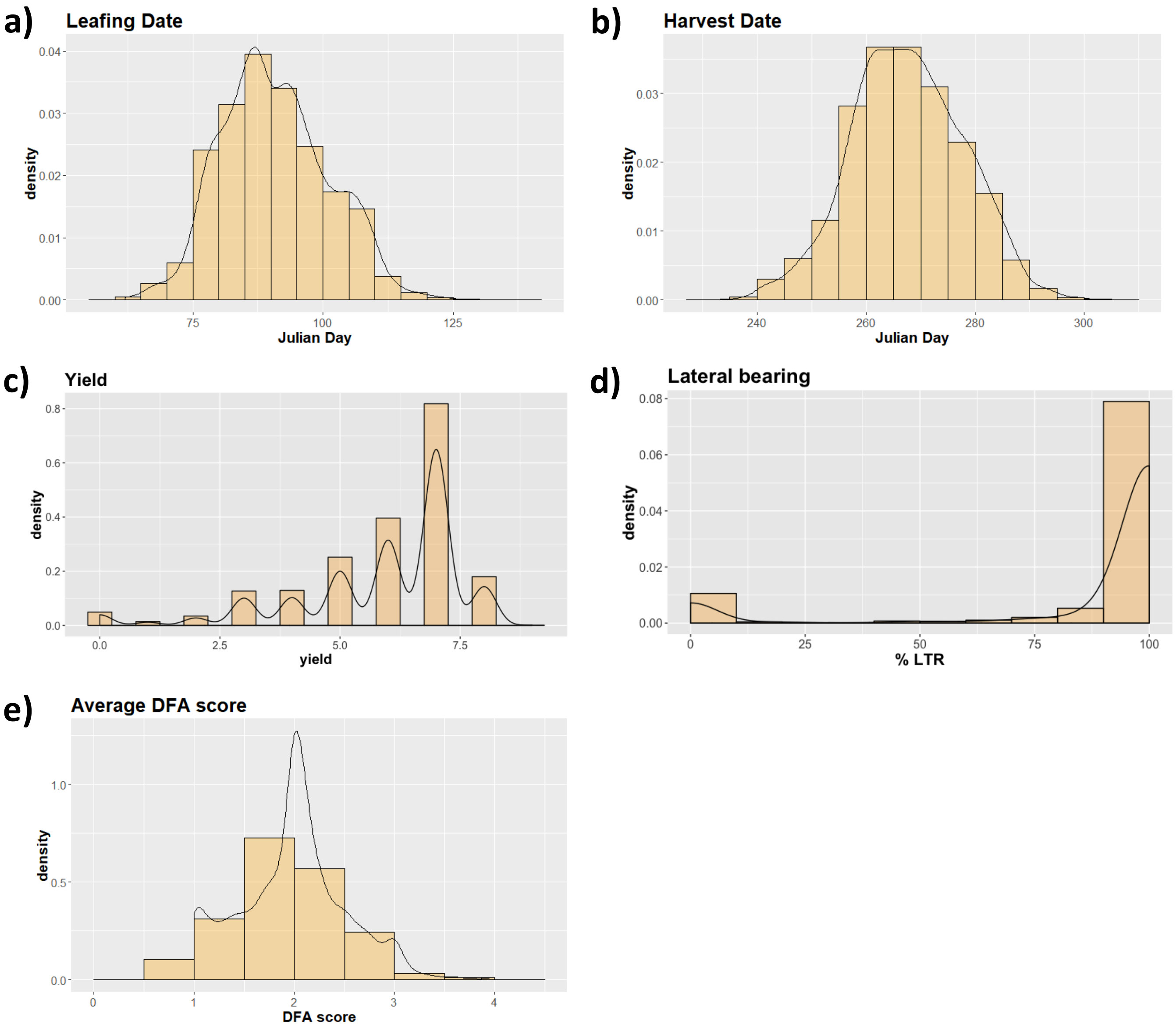

Supplementary Figure S1: Phenotypic distribution of leafing date (a), harvest date (b), yield (c), lateral fruit-bearing (d) and pellicle color (e).
