# Supplementary material for: Deciphering of the Genetic Control of Phenology, Yield, and Pellicle Color in Persian Walnut (Juglans regia L.)
Source: Front Plant Sci. 2019 Sep 20;10:1140. doi: 10.3389/fpls.2019.01140 (PMC6764078; doi:10.3389/fpls.2019.01140)
Supplement: Supplementary file 2 [file DataSheet_2.pdf]

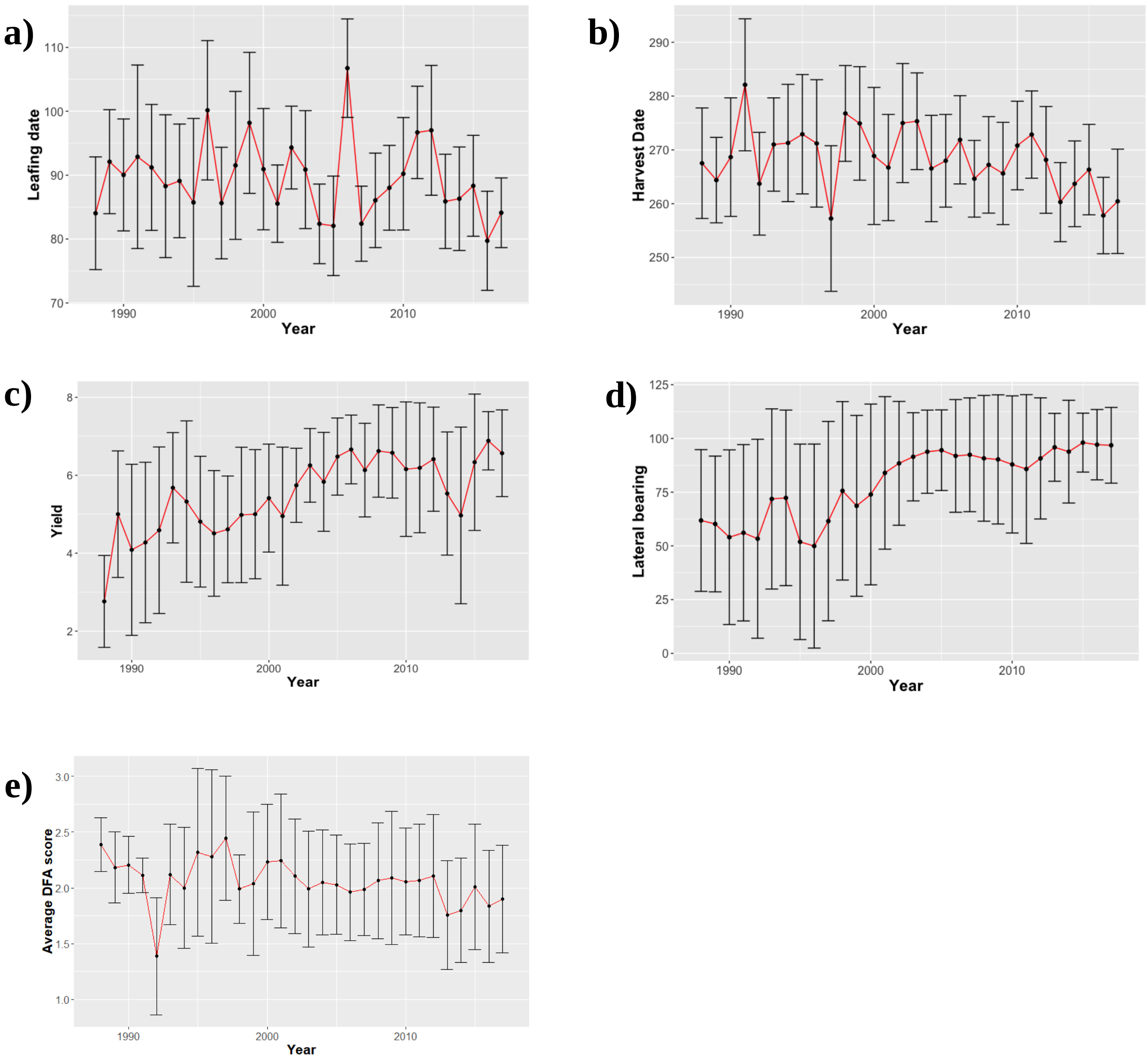

Supplementary Figure S2: Genetic trend of leafing date (a), harvest date (b), yield (c), lateral fruit-bearing (d) and pellicle color (e), within the UCD-WIP from 1988 to 2017.
