# Supplementary figures and images for: Deciphering of the Genetic Control of Phenology, Yield, and Pellicle Color in Persian Walnut (Juglans regia L.)
Source: Front Plant Sci. 2019 Sep 20;10:1140. doi: 10.3389/fpls.2019.01140 (PMC6764078; doi:10.3389/fpls.2019.01140)

**Genetic map**

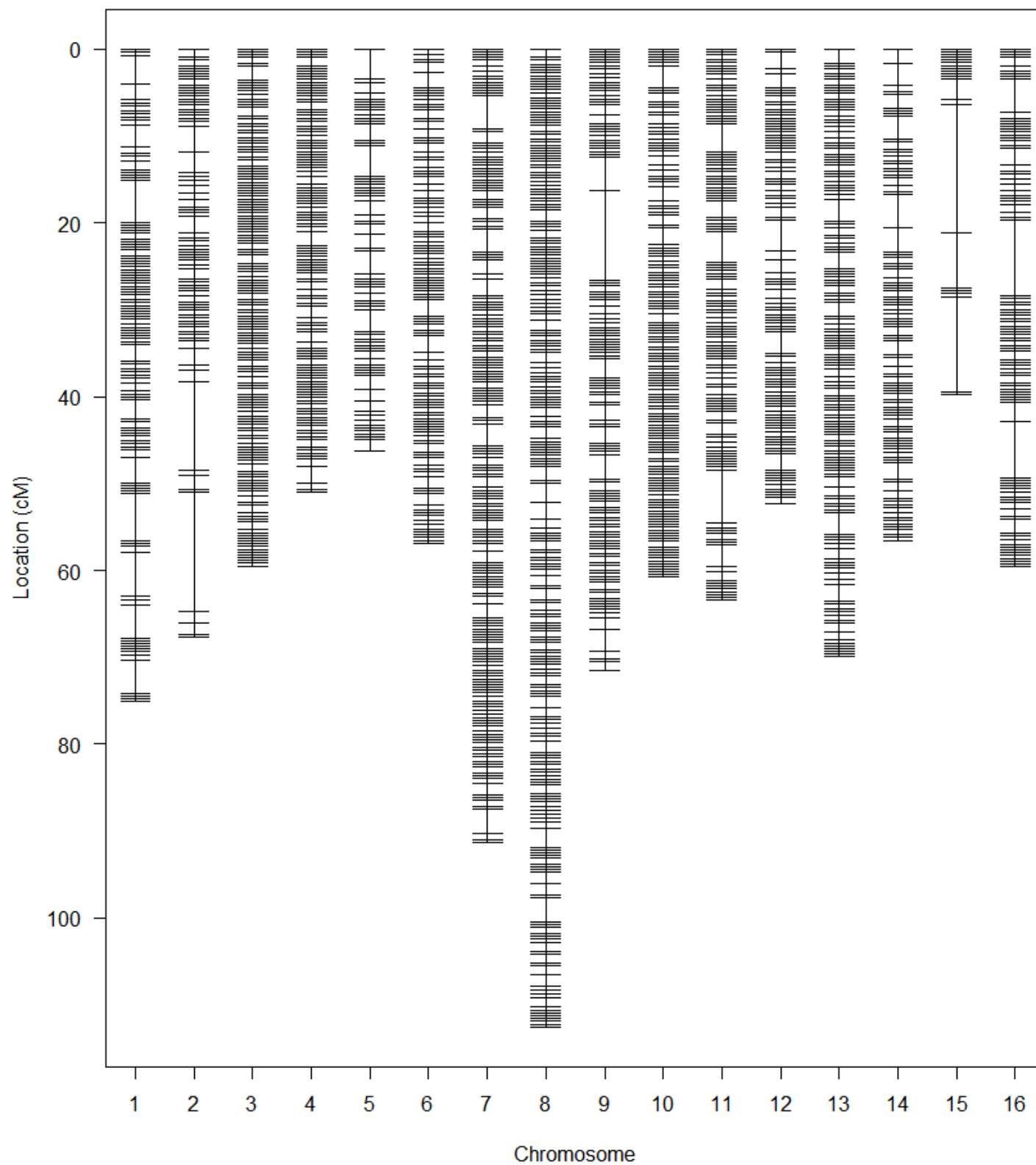

**Supplementary Figure S3.** Genetic map of Chandler.

Supplement: Supplementary file 3 [file DataSheet_3.pdf]

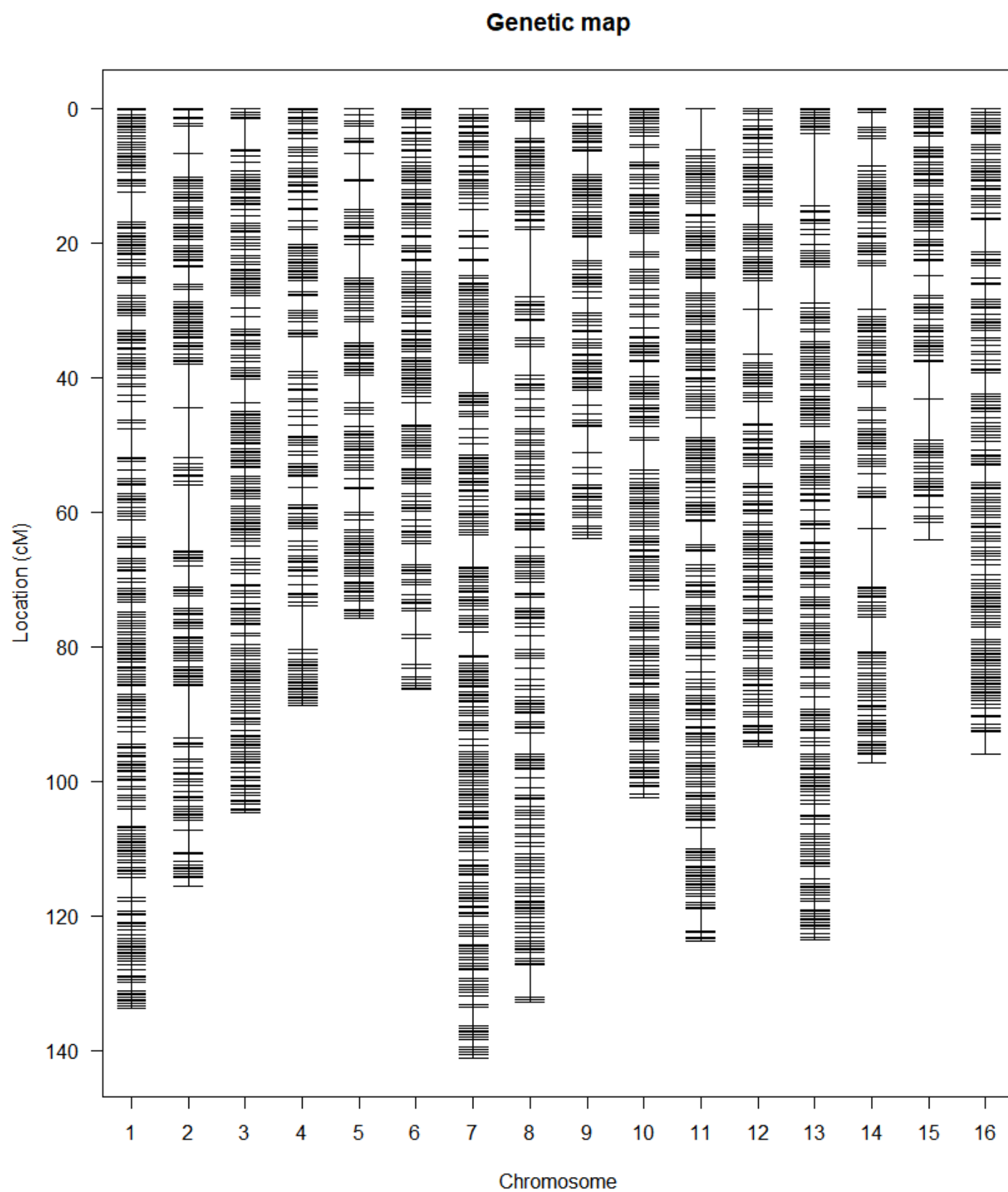

**Supplementary Figure S4.** Genetic map of Idaho.

Supplement: Supplementary file 4 [file DataSheet_4.pdf]

CR LG11

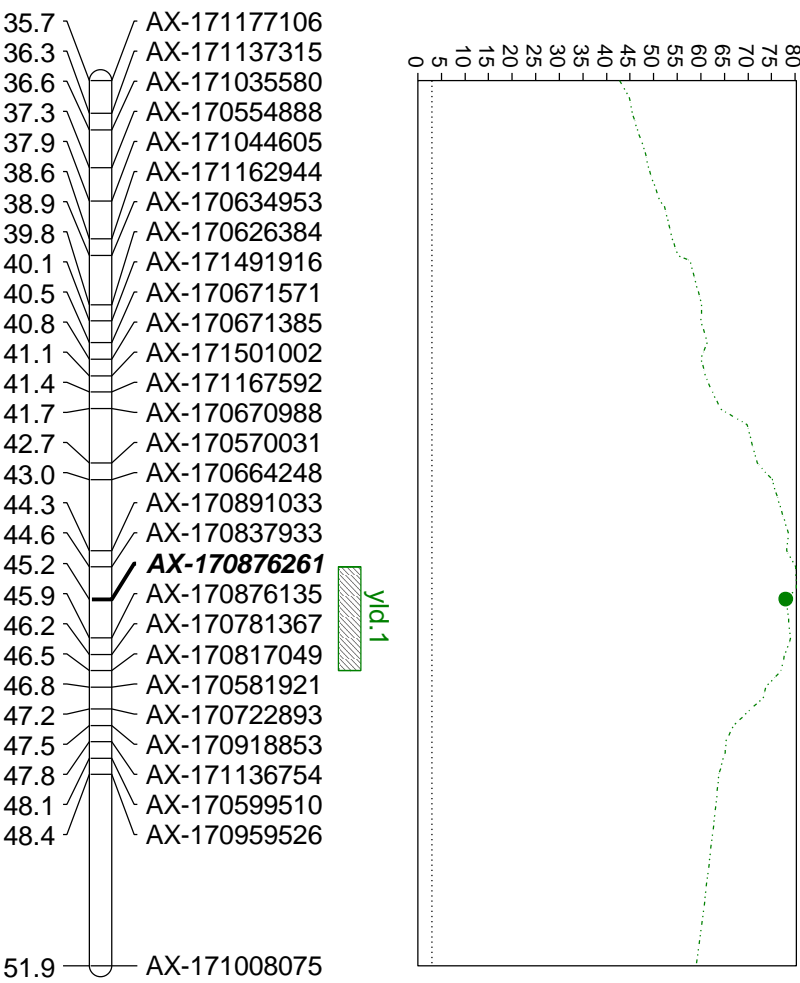

CR LG1

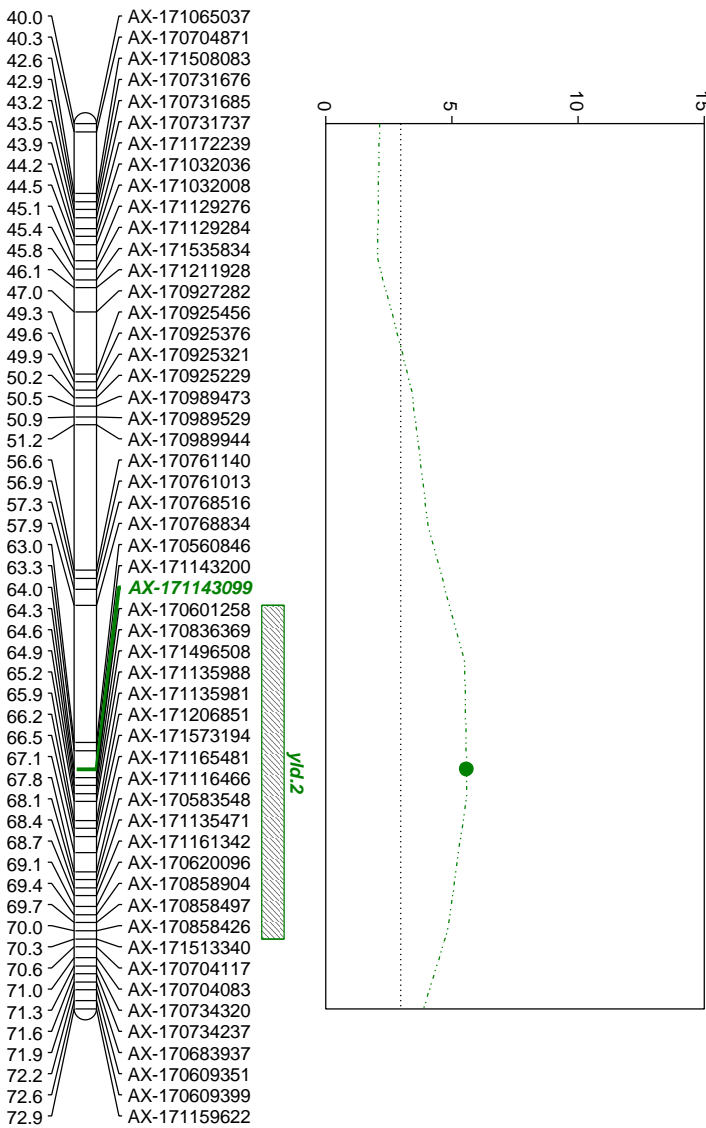

Supplementary Figure S8. QTLs detected for yield (yld) in Chandler (CR).

Supplement: Supplementary file 8 [file DataSheet_8.pdf]

CR LG6

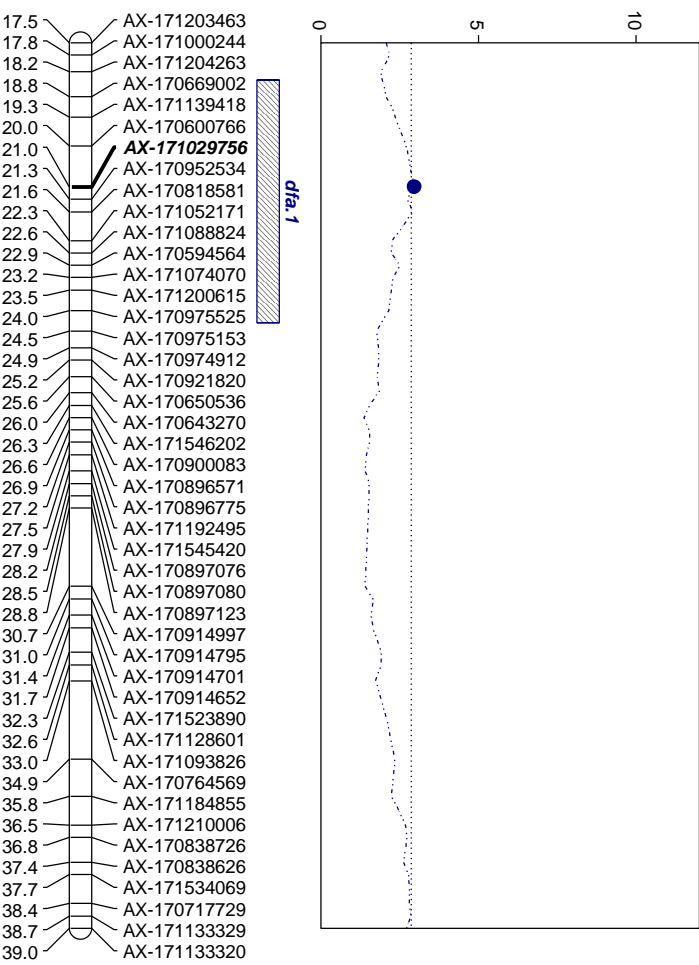

CR LG7

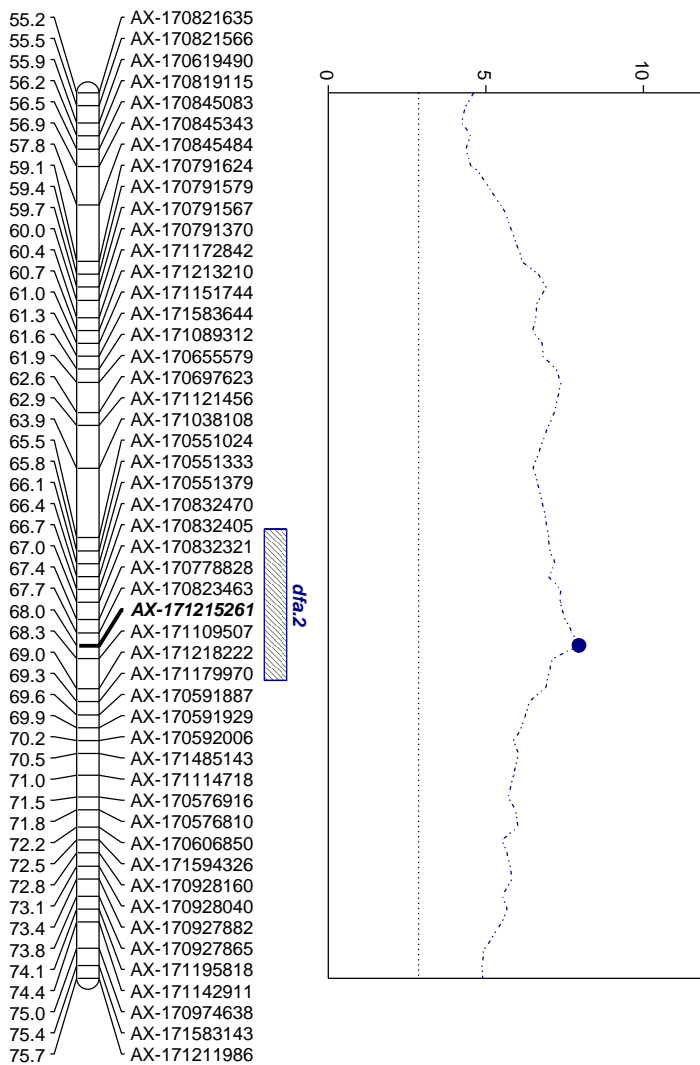

Supplementary Figure S9. QTLs detected for the average DFA score in Chandler (CR).

Supplement: Supplementary file 9 [file DataSheet_9.pdf]

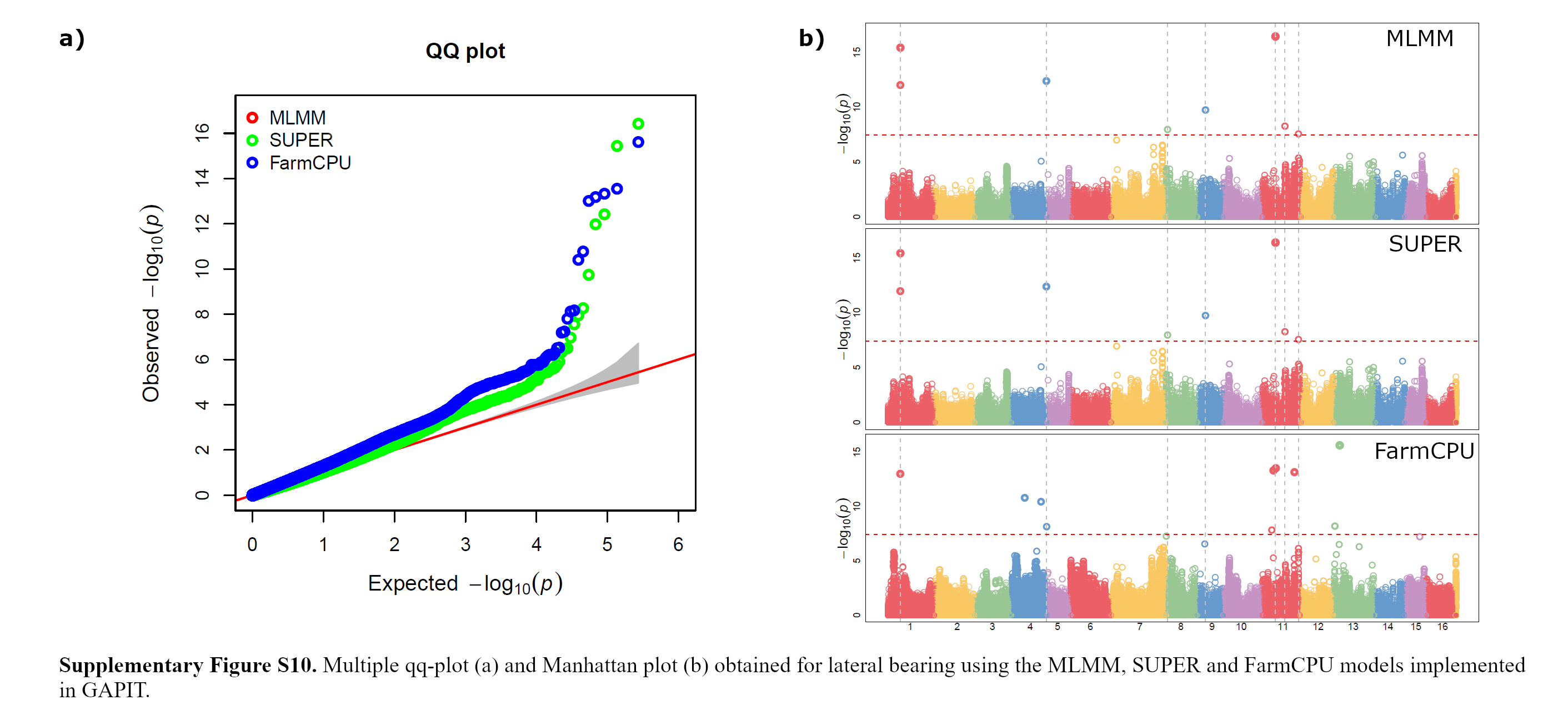

Supplement: Supplementary file 10 [file Image_1.jpeg]

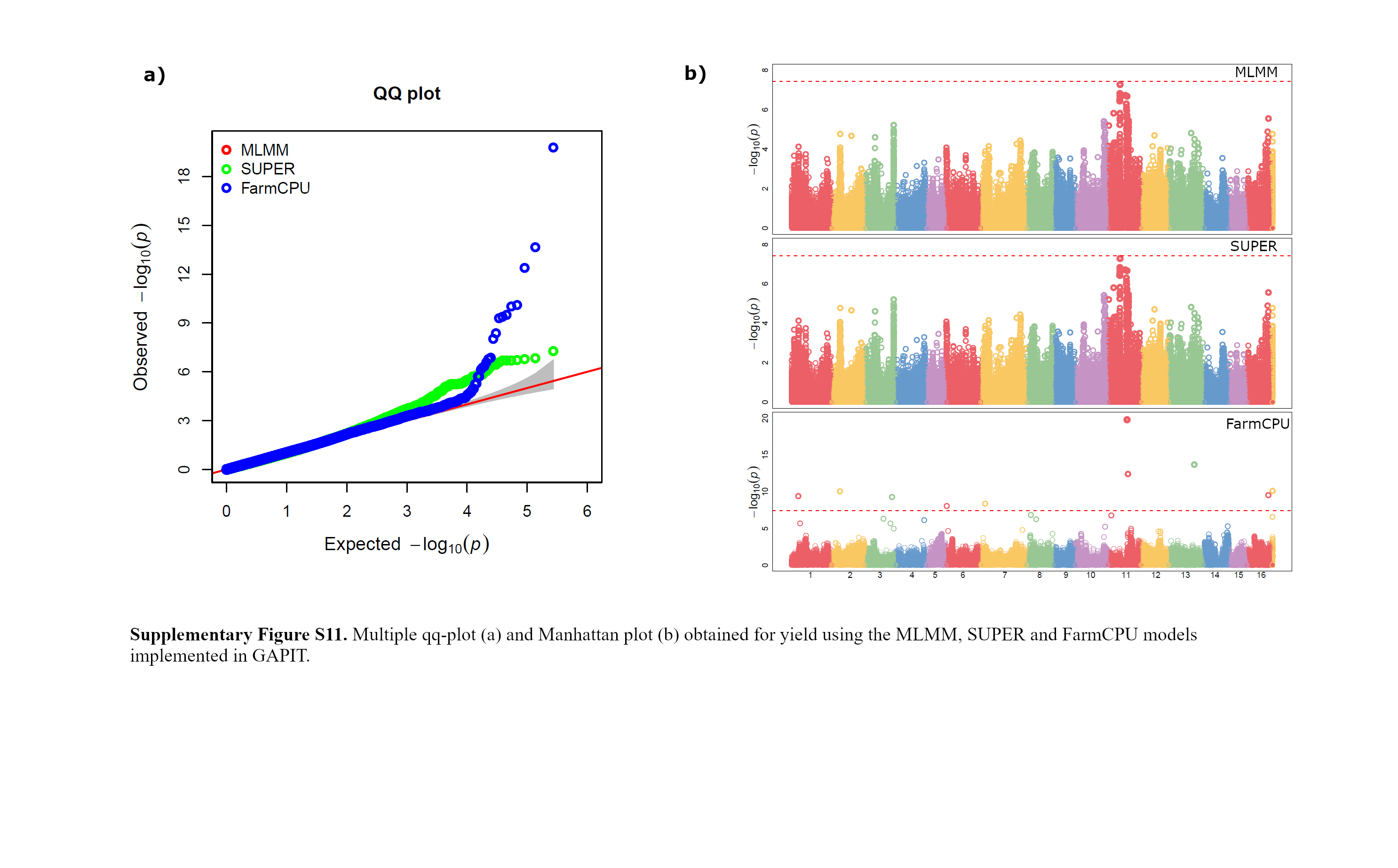

Supplement: Supplementary file 11 [file Image_2.jpeg]

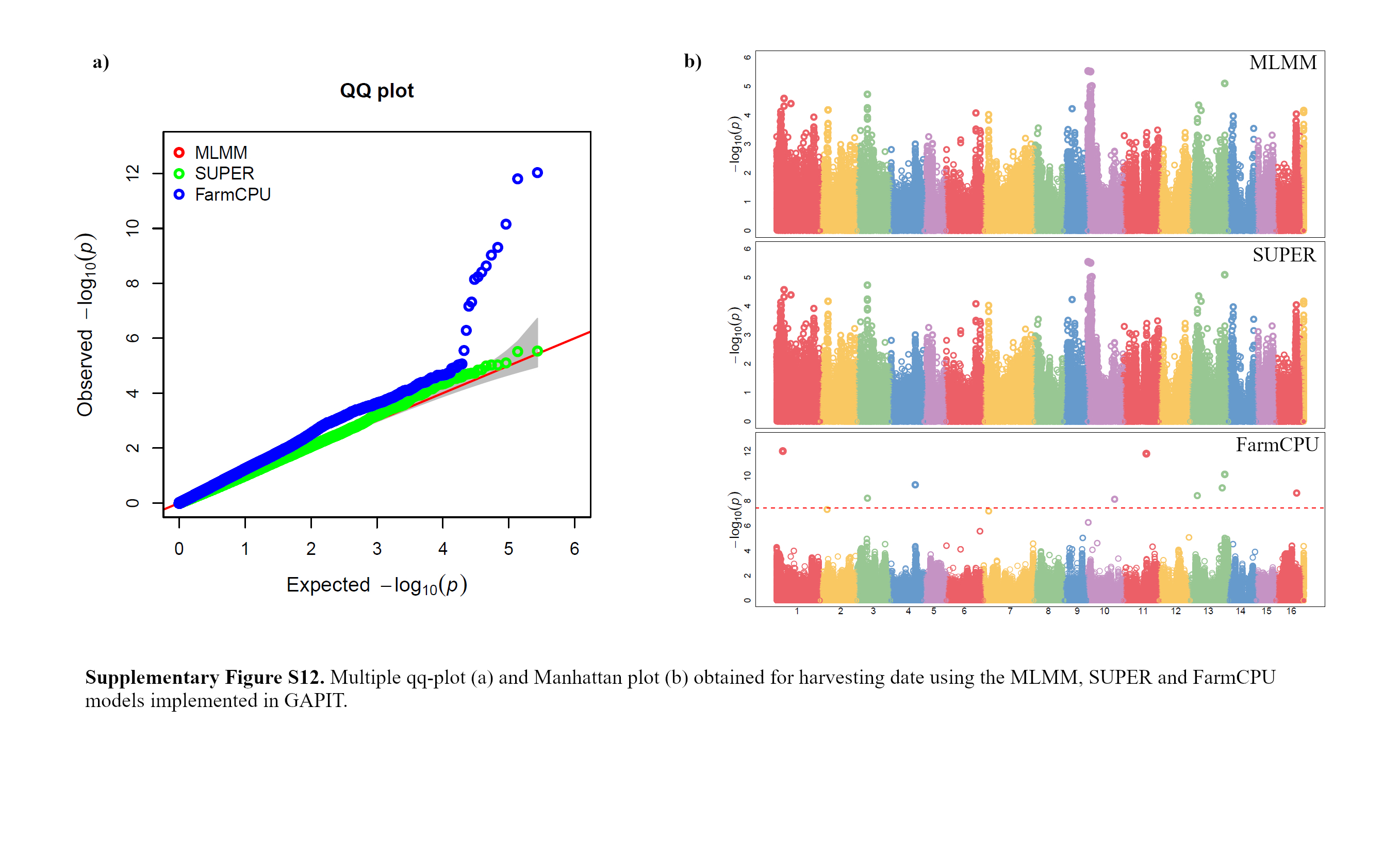

Supplement: Supplementary file 12 [file Image_3.jpeg]

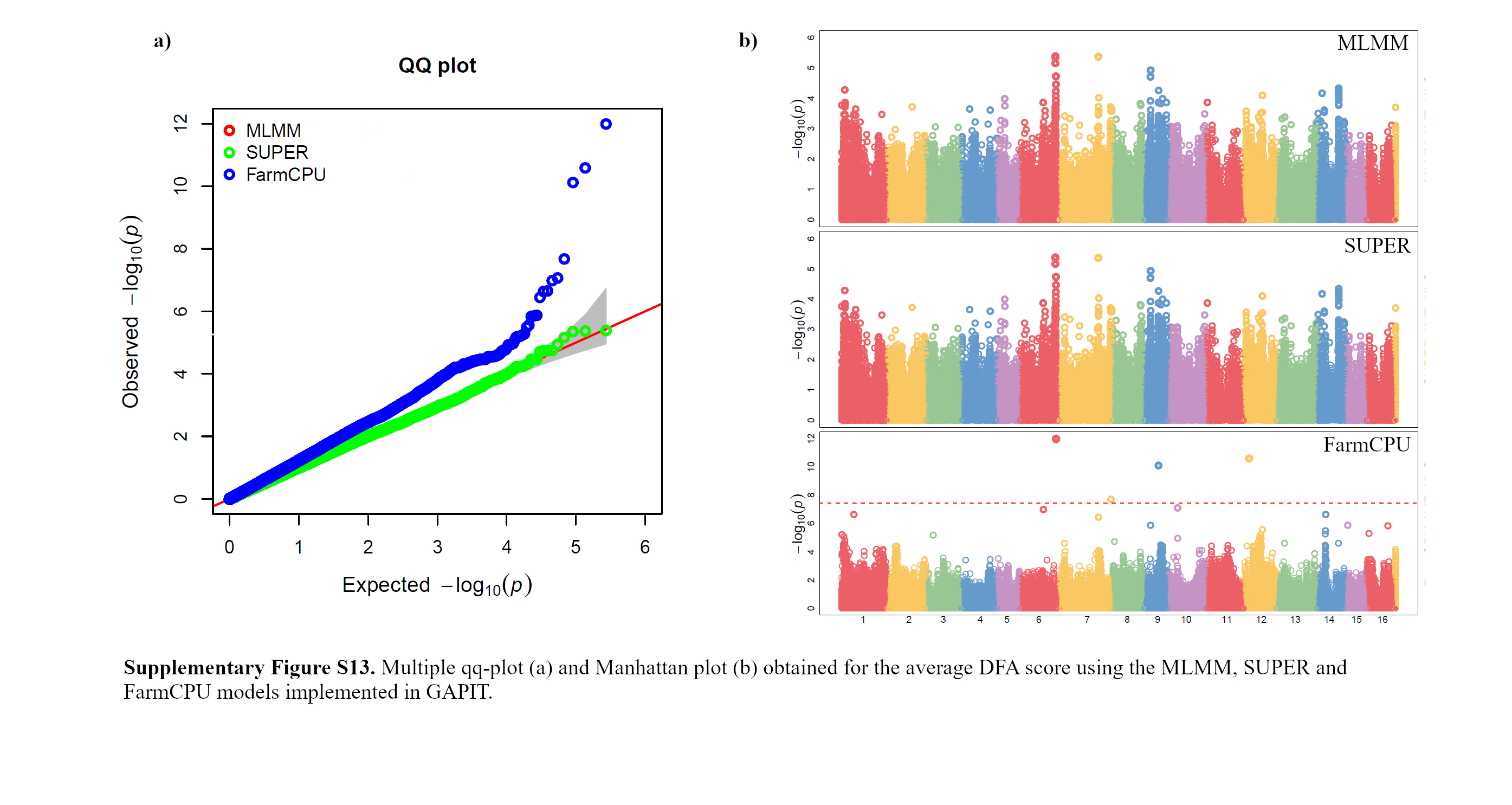

Supplement: Supplementary file 13 [file Image_4.jpeg]

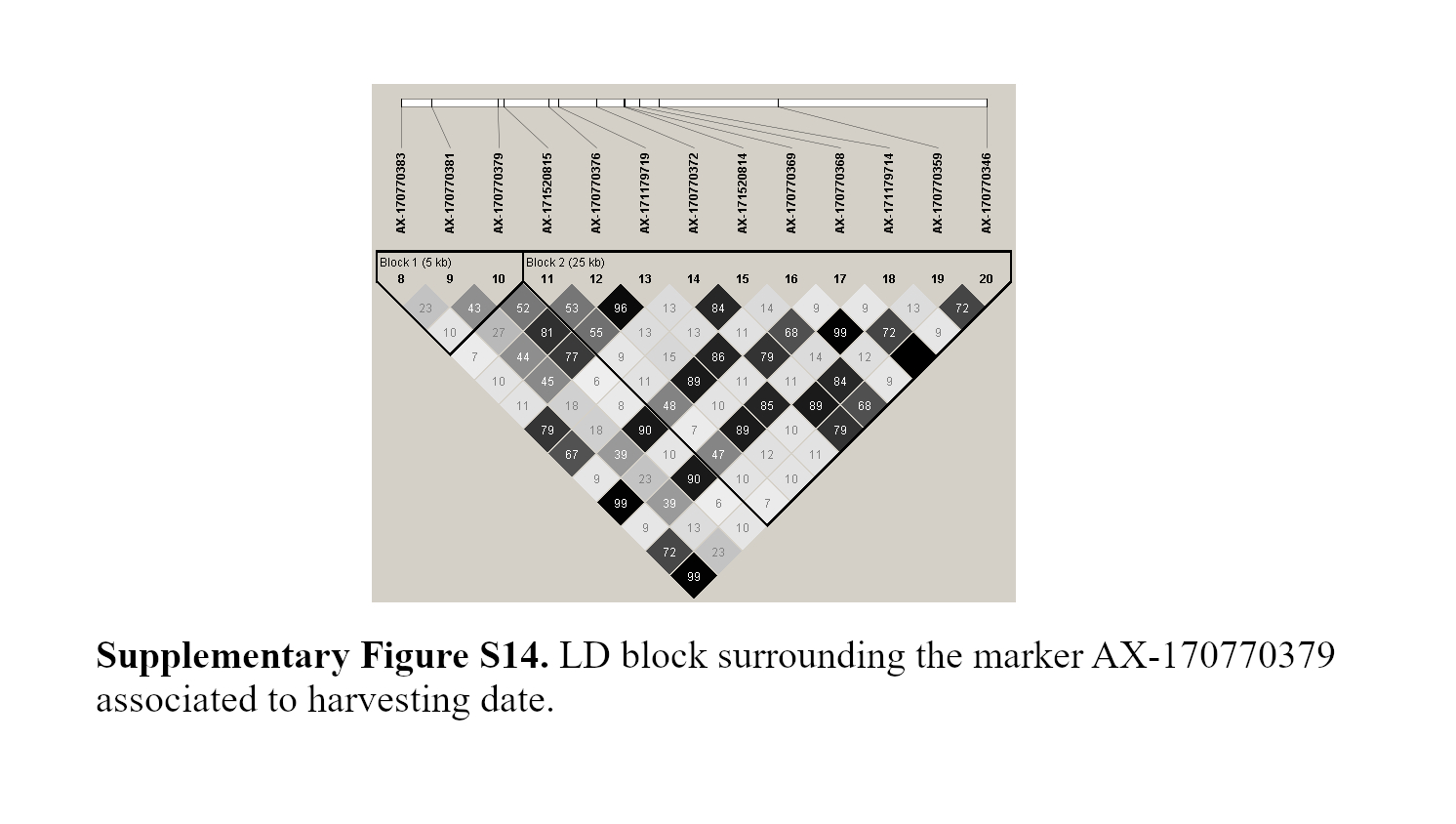

Supplement: Supplementary file 14 [file Image_5.jpeg]

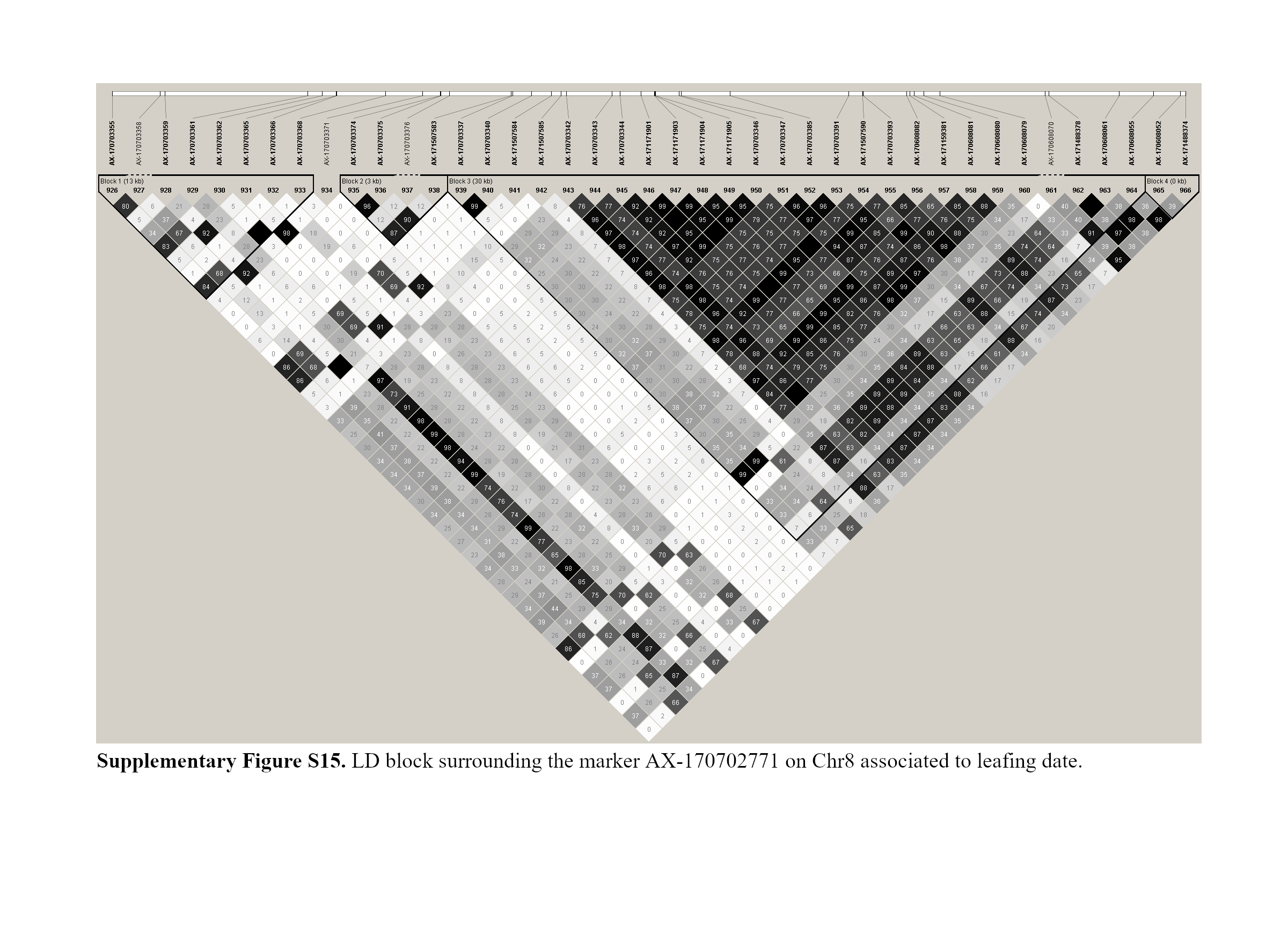

Supplement: Supplementary file 15 [file Image_6.jpeg]

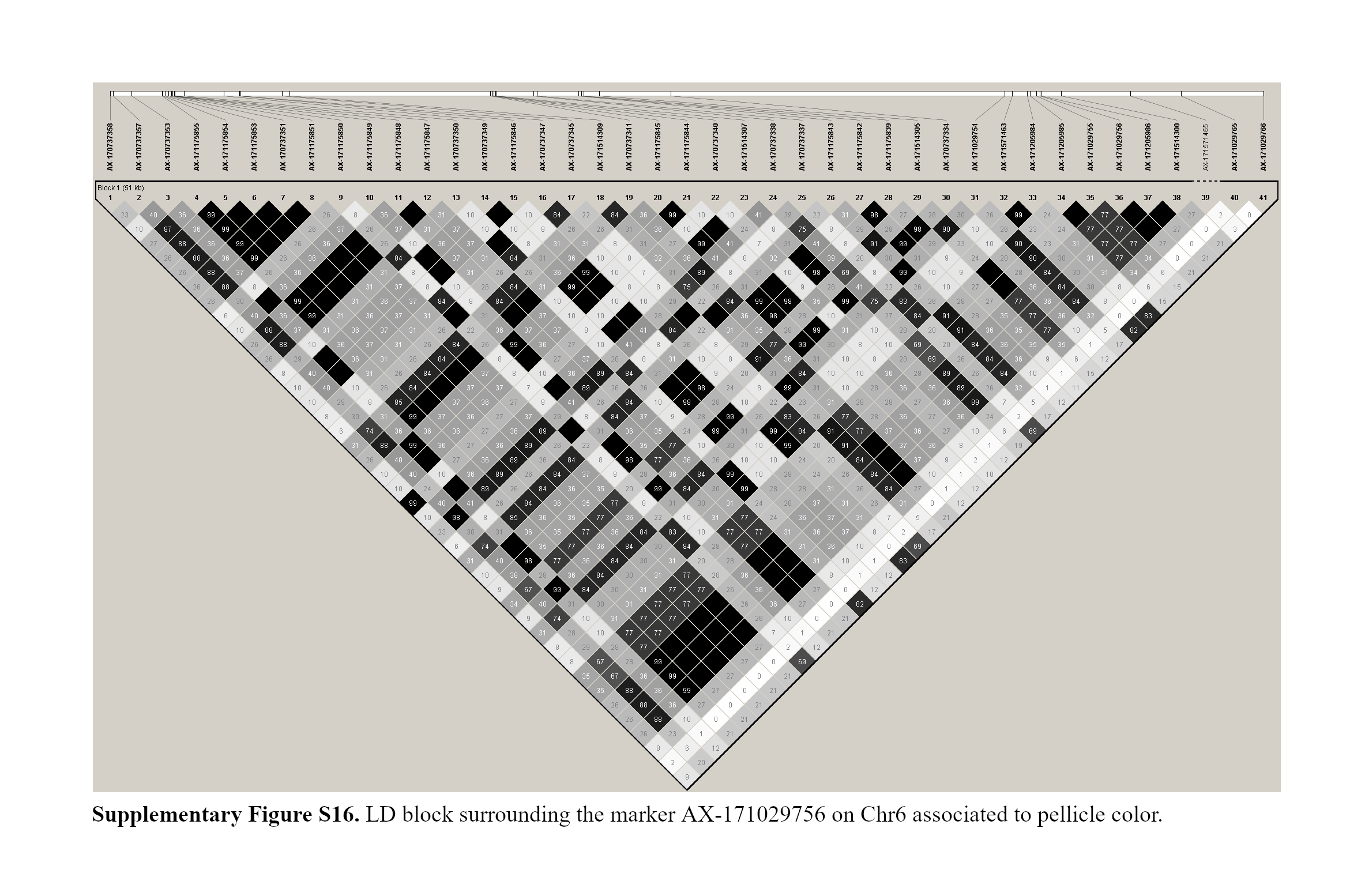

Supplement: Supplementary file 16 [file Image_7.jpeg]
