# Supplementary material for: Deciphering of the Genetic Control of Phenology, Yield, and Pellicle Color in Persian Walnut (Juglans regia L.)
Source: Front Plant Sci. 2019 Sep 20;10:1140. doi: 10.3389/fpls.2019.01140 (PMC6764078; doi:10.3389/fpls.2019.01140)
Supplement: Supplementary file 5 [file DataSheet_5.pdf]

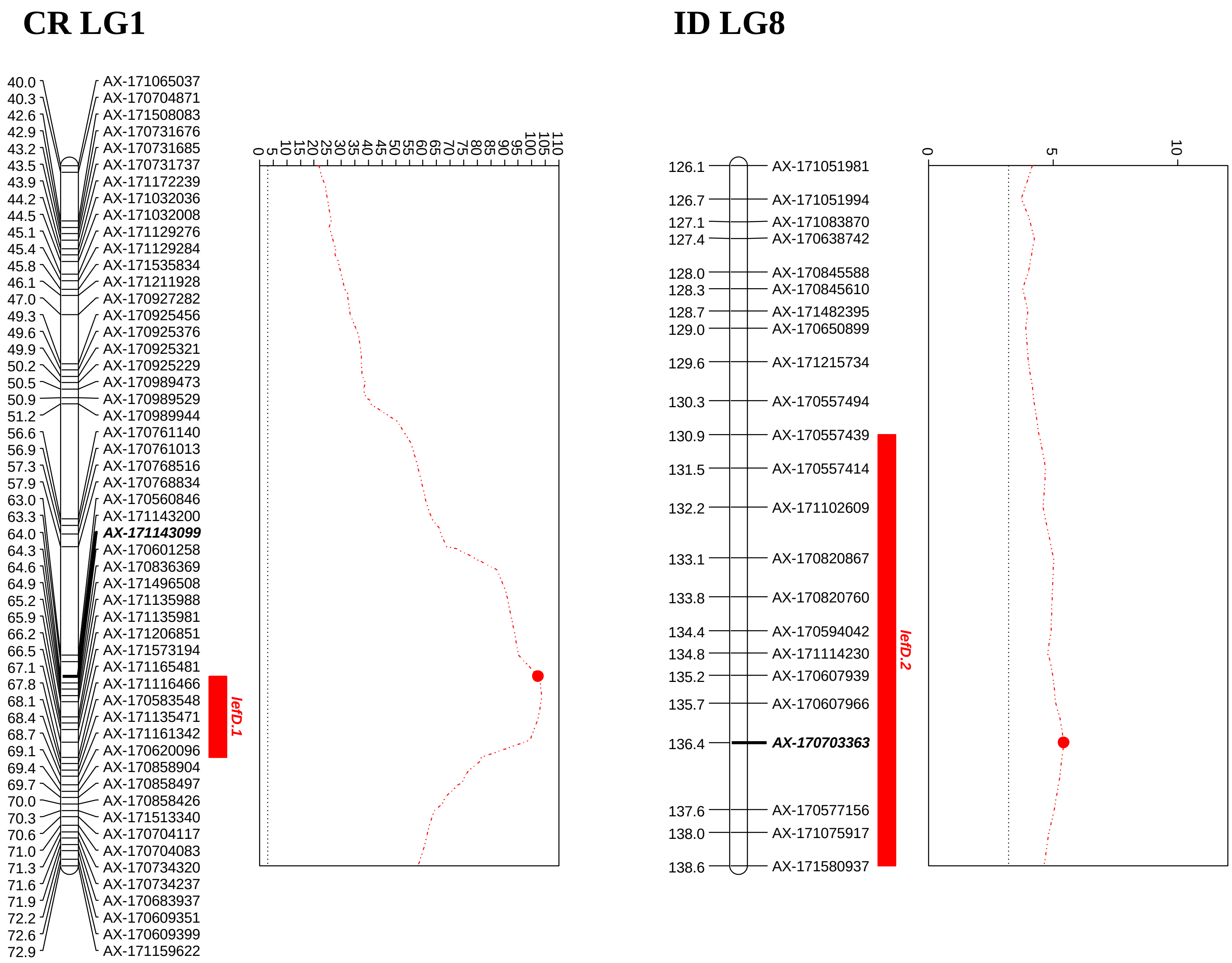

Supplementary Figure S5. QTLs detected for leafing date (lefD) in Chandler (CR) and Idaho (ID)
