# Supplementary material for: Deciphering of the Genetic Control of Phenology, Yield, and Pellicle Color in Persian Walnut (Juglans regia L.)
Source: Front Plant Sci. 2019 Sep 20;10:1140. doi: 10.3389/fpls.2019.01140 (PMC6764078; doi:10.3389/fpls.2019.01140)
Supplement: Supplementary file 7 [file DataSheet_7.pdf]

CR LG11 **ID LG6**

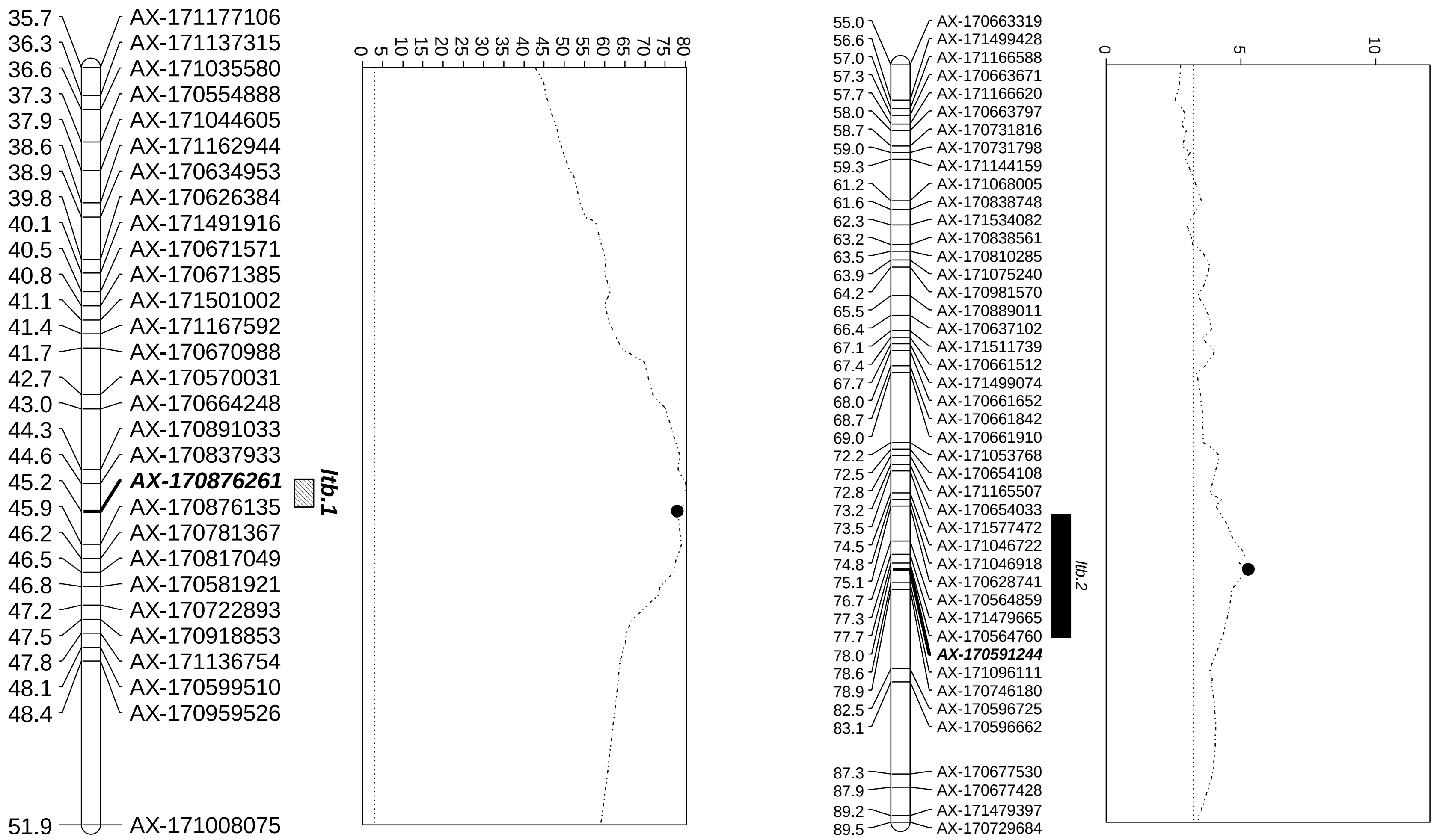

Supplementary Figure S7. QTLs detected for lateral fruit-bearing (ltb) in Chandler (CR) and Idaho (ID)
